# Treatment of cracked teeth: A comprehensive narrative review

**DOI:** 10.1002/cre2.617

**Published:** 2022-07-09

**Authors:** Angeliki Kakka, Dimitrios Gavriil, John Whitworth

**Affiliations:** ^1^ Dental School National and Kapodistrian University of Athens Athens Greece; ^2^ Private Practice Athens Greece; ^3^ MClinDent Restorative Dentistry Newcastle University Newcastle upon Tyne UK; ^4^ Private Practice Korinthos Greece; ^5^ Newcastle University Newcastle upon Tyne UK

**Keywords:** composite resins, cracked tooth syndrome, dental onlay, dental restoration failure

## Abstract

**Objectives:**

The term “cracked tooth” is used to describe an incomplete fracture initiated from the crown and progressing towards a subgingival direction. Despite the high prevalence of cracked teeth and their frequent association with symptoms and pulpal or periapical pathoses, there is still no consensus in the literature with regard to their restorative and endodontic management. Therefore, the aim of this narrative review was to evaluate the most relevant research and provide an up‐to‐date comprehensive overview regarding the treatment of cracked teeth.

**Materials and Methods:**

An electronic literature search was carried out in MEDLINE (via Ovid), Embase (via Ovid), Scopus, and Web of Science as well as several “Grey literature” sources up to February 22nd 2022 using a combination of pre‐specified ‘free‐text' terms (keywords) and “subject headings.” The search process was supplemented by handsearching in relevant dental journals and reference lists. This narrative review focused on clinical follow‐up studies (observational or interventional studies, case series/reports), laboratory studies and systematic reviews written in English language that reported data on treatment of permanent cracked teeth. The selection of relevant studies was carried out by two reviewers (AK and DG) working independently in two consecutive stages: title/abstract screening and full‐text retrieval. Any discrepancies in the study selection were resolved by discussion between the reviewers.

**Results:**

In total, 64 articles were selected for inclusion in this narrative review.

**Conclusions:**

Cracked teeth with normal pulp or reversible pulpitis have exhibited high pulp and tooth survival rates by the provision of direct or indirect composite restorations. Besides, recent data favour monitoring, especially in the absence of symptoms or compromised tooth structure. When endodontic intervention is required, current evidence suggests that along with appropriate restorative management, outcomes of cracked teeth may be comparable to those of non‐cracked root filled teeth.

## 1 INTRODUCTION

The term “cracked tooth” is used to describe an incomplete fracture initiated from the crown and progressing towards a subgingival direction (Rivera & Walton, 2008). “Cracked tooth” comprises one out of the five types of longitudinal tooth fractures, as classified by the American Association of Endodontists (Rivera & Walton, 2008) (the rest are “craze lines,” “fractured cusp,” “split tooth,” and “vertical root fracture”) and is considered the most common (Kim et al., 2020; Seo et al., 2012) and having the most variable prognosis (Rivera & Walton, 2008).

The prevalence of cracked teeth may be high in the adult population. A practice‐based study demonstrated that 70% of patients presented with at least one posterior tooth with visible cracks (Hilton et al., 2011). A greater incidence of cracks has generally been found in mandibular molars (Kim et al., 2013; Krell & Caplan, 2018; Krell & Rivera, 2007). Nevertheless, some Korean studies have reported higher prevalence of cracks in maxillary molars likely because of the altered cusp‐fossa relationship induced by the lingual tilt of their lower antagonists in the Korean population (Roh & Lee, 2006; Seo et al., 2012).

About 20% of cracked teeth may be symptomatic (Hilton et al., 2011), although symptoms are not pathognomonic and could diverge considerably, including pain on biting (Homewood, 1998; Roh & Lee, 2006), sensitivity to cold (Hilton et al., 2018), spontaneous pain (Hilton et al., 2018; Ritchey et al., 1957), tenderness to percussion (Lee et al., 2021a) and even symptoms mimicking orofacial pain, headaches, and trigeminal autonomic cephalalgia (Brynjulfsen et al., 2002; Noma et al., 2017). The etiology of symptoms may be twofold: dentinal fluid movement due to separation of cracked segments under load (Davis & Overton, 2000) and pulpal or periapical pathoses induced by bacteria and their by‐products, which penetrate crack lines (Ricucci et al., 2015). Therefore, the term “cracked tooth syndrome,” which was proposed to describe the common symptoms associated with cracked teeth (Cameron, 1964), has been characterized as rather misleading; a crack should not be viewed as a disease on its own, but as a potential cause of pulpal and periradicular diseases (Abbott & Leow, 2009). In fact, the diagnosis of cracked tooth was confirmed in only 5.6% of teeth suspected of having cracks according to their symptoms (Kang et al., 2016).

The importance of early diagnosis of cracked teeth should be highlighted. Delayed diagnosis has been linked to increased rate of pulpal complications (Kang et al., 2016; Kim et al., 2013) while any associated bone defects could complicate future implant placement in case of eventual tooth loss (Dutner et al., 2020). Diagnosis of cracked teeth can be confirmed through various methods, including visual inspection under magnification (Clark et al., 2003), staining (Abou‐Rass, 1983), transillumination (Kim et al., 2020), bite tests (Seo et al., 2012; Yang et al., 2019), autofluorescence (Jun et al., 2019), optical coherence tomography (Shimada et al., 2020), quantitative percussion diagnostics (Sheets et al., 2020), and lasers (Sapra et al., 2020). As for radiographic methods, cone beam computed tomography is considered superior to periapical radiographs in depicting the extent of cracks (Wang et al., 2017), however, this might still be of limited value especially in endodontically treated teeth (PradeepKumar et al., 2021).

With regard to the treatment of cracked teeth, there is still no consensus in the literature. Management approaches vary according to baseline pulpal diagnosis, which often determines the need for endodontic intervention (Kim et al., 2013), whereas considerable variation has also been noted within cohorts with similar pulpal diagnoses. That was depicted by recent questionnaire‐based surveys (Alkhalifah et al., 2017; Yap et al., 2021), which showed large differences in the treatment approaches among prosthodontists, endodontists and general practitioners, both as groups and within each group, especially for cases without symptoms. For instance, the recorded managements of a minimally restored asymptomatic vital cracked premolar involved a full crown (around 35% of participants), endodontic treatment followed by crown (22%), monitoring (20%), and extraction (17%) (Alkhalifah et al., 2017). Conflicting views have also been observed pertaining to the management of cracked teeth with pulpal involvement. Berman and Kuttler (2010) pointed out that teeth with pulp necrosis due to the presence of cracks have poor prognosis and should be considered non‐restorable. On the contrary, a prospective study that included both cracked and noncracked teeth found that preoperative presence of cracks was not a significant prognostic factor for tooth loss after primary or secondary endodontic treatment (Ng et al., 2011a). It was also demonstrated that endodontists were less likely inclined to extract a tooth with deep cracks and pulpal involvement compared to prosthodontists and general dental practitioners (Alkhalifah et al., 2017; Yap et al., 2021).

Given the wide variation of treatment approaches and the lack of specific guidelines, the aim of this article was to provide a comprehensive up‐to‐date overview pertaining to the treatment of cracked teeth. Whilst systematic reviews are considered as the most rigorous method to synthesize the evidence base with regard to a specific topic, the broad scope of the subject as well as the substantial heterogeneity of the relevant literature would preclude reliable synthesis of the relevant data and this could lead to the exclusion of important findings. Therefore, a comprehensive narrative review was deemed preferable.

## 2 METHODOLOGY OF THE REVIEW

An electronic literature search was carried out in the databases MEDLINE (via Ovid), Embase (via Ovid), Scopus, and Web of Science up to March 27, 2021 with no limits for the date of publication while additional searches were performed in September 21, 2021 and February 22, 2022. Several trial registers or “Grey literature” sources were also searched to ensure a thorough coverage of the subject. A combination of “free‐text” terms (keywords) and “subject headings” was used. The following terms were searched: “cracked tooth/teeth,” “incompletely fractured tooth/teeth,” “incomplete tooth fracture/fractures,” “incomplete coronal fracture/fractures,” “incomplete crown fracture/fractures,” “tooth crack/cracks,” “longitudinal tooth fracture/fractures,” “longitudinally fractured tooth/teeth,” “cracked tooth syndrome.” Additionally, handsearching was performed in relevant dental journals and reference lists of the articles retrieved.

For this narrative review, the authors focused on utilizing the findings of clinical follow‐up studies (observational or interventional studies, case series/reports), laboratory studies, and systematic reviews written in English language that reported data on treatment of permanent cracked teeth. Study selection was performed by two reviewers (AK and DG) working independently in two consecutive stages: screening of titles/abstracts and retrieval of full texts. Any discrepancies were resolved by discussion between the reviewers.

**Figure 1:**
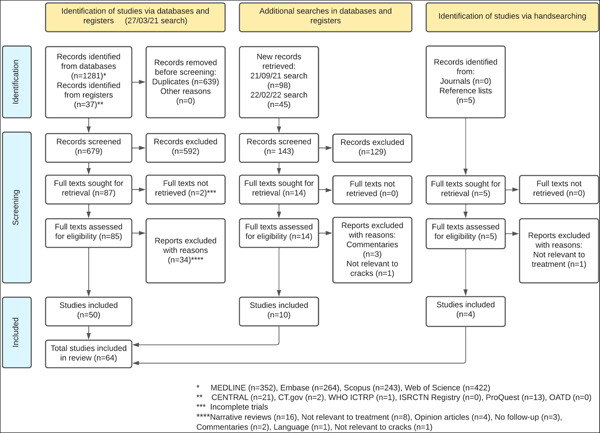
Study selection process illustrated in a PRISMA flow diagram.

The study selection process is illustrated in a PRISMA flow diagram (Page et al., 2021) (Figure 1). The initial electronic search provided 1318 results. After removal of duplicates, 679 titles and abstracts were screened and full texts were obtained for 85 articles. Finally, 50 studies were accepted for inclusion. The same process was followed for the two additional electronic searches leading to the inclusion of 10 further studies and the handsearching in journals and reference lists, which provided four studies. In total, 64 articles were selected for inclusion in this narrative review. The majority were observational clinical studies (*n* = 35) followed by case series/reports (*n* = 17), in vitro studies (*n* = 6), interventional clinical studies (*n* = 4), and systematic reviews (*n* = 2). Lists of the 64 included studies as well as the studies excluded at full‐text stage (along with the reasons for exclusion) are provided in Online Supporting Information of this article.

Based on the diagnosis of pulpal involvement, the 64 included studies could be also classified into three main categories (eight studies were included into both first and second categories since they provided relevant information for both aspects):

1) Studies relevant to the treatment of cracked teeth with normal pulp or reversible pulpitis (*n* = 30) (Tables 1 and 4).
2) Studies relevant to the treatment of cracked teeth requiring endodontic treatment or being previously endodontically treated (*n* = 34) (Tables 2 and 4).
3) Studies including cracked teeth with mixed or unspecified pulpal diagnoses and treatments (*n* = 9) (Table 3).

**Table 1:** Clinical studies relevant to restorative approaches for cracked teeth with baseline normal pulp or reversible pulpitis

Study	No of teeth	Management of crack lines	Interim treatment (for multiple‐stage approaches)	Definitive treatment	Follow‐up	Outcome
**Interventional studies (*n*** **=** **3)**						
Davis and Overton (2000)	40	N/S^+^	N/A^+^	Bonded amalgam (20 teeth), pin‐retained amalgam (20 teeth)	1 year	Bite pain resolved for both groups, cold sensitivity resolved only for bonded group
Opdam and Roeters (2003)	40	N/S	N/A	Direct composite with (20) or without (20) cuspal coverage	6 months –preliminary report of Opdam et al. (2008)	Only 50% of teeth symptom‐free, no significant difference between groups
Opdam et al. (2008)	41	N/S	N/A	Direct composite with (21) or without (20) cuspal coverage	7 years	93% pulp survival (no significant difference between groups), higher failure rate for restorations without cuspal coverage
**Observational studies (*n*** **=** **15)**						
Abbott (2001)	100	Complete crack elimination	Sedative liner and glass ionomer restoration	N/A	3 months – preliminary report of Abbott and Leow (2009)	81% pulp survival, 100% tooth survival
Abbott and Leow (2009)	100	Complete crack elimination	Sedative liner and glass ionomer restoration	Crowns or onlays	Up to 5 years	80% pulp survival (recall rate was 54%), 100% tooth survival
Banerji et al. (2014)	151	N/S	Supra‐coronal direct composite splint (DCS)	Direct (74%) or indirect (1.6%) composite onlays, gold onlays (10%), crowns (14%)	3 months (for DCS), no follow‐up for definitive restorations	93% pulp survival, another 5% had DCS failure or intolerance
Chana et al. (2000)	6	N/S	N/A	Metal onlays	4 years	100% pulp and tooth survival
de Toubes et al. (2022)^±^	26	N/S	Direct composite	Crowns or onlays	3.3 years	88% pulp survival, 1 tooth was lost after endodontic treatment
Guthrie and DiFiore (1991)	28	N/S	Provisional crown	Crowns	1 year (definitive), 2 weeks (interim)	89% pulp survival
Homewood (1998)	62	N/S	Orthodontic bands (8 teeth initially and 3 further teeth after symptoms persistence with definitive treatment)	Single‐stage: Amalgam or composite cuspal coverage or crowns,	15 months	94% overall pulp survival (83% for teeth that received orthodontic bands), 98% tooth survival
				Multiple‐stage: Crowns or onlays,		
				1 tooth extracted		
Kanamaru et al. (2017)^±^	44	N/S	Occlusal adjustment (25 cases)	For pulp‐preserved group: Crowns (70.4%), occlusal adjustment (14.8%), composite resin (7.4%), monitoring (7.4%)	1–3 years	61% overall pulp survival (pulp complications occurred before definitive restoration)
			Eugenol‐sedation (9 cases)			
			Resin coating for dentine hypersensitivity (3 cases)			
			Restoration (2 cases)			
			Monitoring (5 cases)			
Kang et al. (2016)^±^	58	N/S	Provisional crowns (38 teeth)	Crowns (27 teeth from those that received provisional crowns), direct composite (10 teeth), inlays (10 teeth)	Not specified	71% pulp survival after interim treatment
Kim et al. (2013)^±^	21	N/S	Provisional crowns	Crowns	Not specified	58% pulp survival after interim treatment
Krell and Rivera (2007)	127	Previous restorations and cracks were not removed	N/A	Crowns	6 months	79% pulp survival
Lee et al. (2021a)	29	Cracks were removed until a shallow crack remained close to the pulp and were lined the crack with flowable composite	Bidirectional splinting	Crowns	2.6 years	72% pulp survival (91% after definitive restoration), 100% tooth survival
Marchan et al. (2013)	6	N/S	N/A	Metal onlays	3.5 years	100% pulp and tooth survival
Signore et al. (2007)	43	N/S	N/A	Indirect composite onlays	6 years	93% pulp survival
Wu et al. (2019)	199	N/S	Orthodontic bands	Crowns (18 teeth remained with bands)	3 years (3 months of interim treatment)	71% overall pulp survival (81% 5‐year estimated survival with crowns, 37% with bands), 3 teeth extracted
**Case reports/series (*n*** **=** **9)**						
Batalha‐Silva et al. (2014)	1	Complete crack elimination (after banding phase)	Bidirectional splinting	Direct composite	5 weeks (for interim treatment)	The tooth remained vital and asymptomatic
Bearn et al. (1994)	4	N/S	N/A	Bonded amalgam	15–26 months	All cases remained vital and asymptomatic
de Toubes et al. (2020)^±^	1	Crack lines were disinfected with chlorhexidine	Intra‐coronal direct composite	Crown	5 years	The tooth remained vital
Ehrmann and Tyas (1990)	3	N/S	Orthodontic bands	Crowns	30 months to 14 years	All cases remained vital and asymptomatic
Griffin (2006)	2	Cracks removed until no separation of the tooth could be felt with a sharp probe	N/A	Ceramic onlays	2 years	Both teeth remained vital and asymptomatic
Ito et al. (1998)^±^	2	Cracks partially removed to identify their relation to the pulp	Intra‐coronal direct composite	Crowns	2 years (6 months of interim treatment)	One of the teeth required endodontic treatment after the 6‐month interim treatment, the other tooth remained vital
Liebenberg (1996)	2	Cracks removed until they diminished to a fine craze	N/A	Ceramic onlays	20 months	Both teeth remained vital and asymptomatic
Ritchey et al. (1957)^±^	3	N/S	Zinc‐oxide eugenol liner (case 3)	Crowns (Cases 11, 12)	2 years (Cases 11, 12)	Cases 11,12 remained vital and asymptomatic, Case 3 developed irreversible pulpitis and was extracted due to crack extending to pulpal floor
					3 days (Case 3)	
Yap (1995)	1	N/S	N/A	Metal onlay	1 year	The tooth remained vital and asymptomatic

**● +**: N/S = not specified, N/A = not applicable.

**● ±**: Data from these studies are also included in Table 2 since they provide information regarding treatment of cracked teeth that received endodontic treatment.

**Table 2:** Clinical studies and systematic reviews focusing on cracked teeth that received endodontic treatment

Study	No of cracked teeth	Management of crack lines	Interim treatment (intra‐ or post‐endodontic)	Definitive post‐endodontic restoration	Follow‐up	Outcomes
**Systematic reviews (*n*** **=** **2)**						
Leong et al. (2020)	See below data from included studies (Kang et al., 2016; Krell & Caplan, 2018; Sim et al., 2016; Tan et al., 2006)				5 years (estimated)	84% survival rate
Olivieri et al. (2020)	See below data from included studies (Dow, 2016; Kang et al., 2016; Kim et al., 2013; Krell & Caplan, 2018; Krell & Rivera, 2007; Sim et al., 2016; Tan et al., 2006)				1 year (estimated)	88% survival rate, 82% success
**Interventional study (*n*** **=** **1)**						
Lu et al. (2021)	87	N/S^+^	Temporary filling	Crowns (45 teeth), direct composite (42 teeth)	6 months	Crowns exhibited significantly better therapeutic effect, bite force, chewing efficiency, quality of life as well as reduced periodontal index compared to direct composite restorations
**Observational studies (*n*** **=** **17)**						
Chen et al. (2021)	62	N/S	N/S	Crowns (15 teeth remained with temporary filling)	23.3 months	75.8% overall success rate; 93.6% with crowns; 20% for unrestored teeth
Davis and Shariff (2019)	65	N/S	Occlusal adjustment (post‐endodontic)	Resin composite core (with intra‐orifice barriers) and crowns	2–4 years (mean 2.8)	96.6% survival rate; 90.6% success
de Toubes et al. (2022)^±^	63	N/S	Direct composite or temporary filling; occlusal adjustment was performed for some cases	Crowns or onlays	3.3 years	90.5% survival rate
Dow (2016)	15	N/S	N/S	Not specified	16.2 months	67% survival rate; 46.6% success rate
Kanamaru et al. (2017)^±^	17	N/S	N/S	Crowns	1–3 years	100% survival rate
Kang et al. (2016)^±^	88	N/S	Provisional crowns or orthodontic bands (post‐endodontic)	Crowns	2 years	90% survival rate
Kim et al. (2013)^±^	60	N/S	Provisional crowns (post‐endodontic)	Crowns	2 years	98.3% survival rate
Krell and Caplan (2018)	363	N/S	N/S	Crowns	1 year	82% success rate
Krell and Rivera (2007)^±,§^	14	N/S	N/S	Crowns	1 year	93% survival and success rate
Lee et al. (2021a)^±^	8	See Table 2	Provisional crowns (post‐endodontic)	Crowns	2.6 years	100% survival rate
Liu et al. (2021)	10	N/S	N/S	N/S	19 months	40% success rate
Malentacca et al. (2021)	87	Flowable resin was applied with a size 6 K file under microscope magnification to seal crack lines extending beyond the canal orifices	Occlusal adjustment and pre‐endodontic reconstruction (for heavily compromised teeth)	Direct cuspal coverage composite or crowns	5.6 years	68% 5‐year survival and 53% success rate
Ng et al. (2011a)	127	N/S	N/S	N/S	2–4 years	Survival rate of 95.3% for primary endodontic treatment and 96.8% for endodontic retreatment
Ng et al. (2011b)	199 (counted as the number of roots)	N/S	N/S	N/S	2–4 years	Success rate of 77% for primary endodontic treatment and 76.8% for endodontic retreatment
Nguyen Thi and Jansson (2021)	200	N/S	N/S	Composite restorations (75%), full crowns (24%)	4.5 years	68% 5‐year survival rate (97% with full crowns)
Sim et al. (2016)	84	N/S	N/S	Crowns or amalgam cores + orthodontic bands	5 years	95% survival rate
Tan et al. (2006)	50	Crack lines were left in situ	N/S	Crowns or amalgam cores + orthodontic bands	2 years	85.5% survival rate
**Case reports/series (*n*** **=** **11)**						
de Toubes et al. (2020)^±^	2	See Table 2	Direct composite in infra‐occlusion	Crowns	5‐years	Both teeth survived
Dutner et al. (2020)	1	N/S	N/S	Crown	30 months	The tooth was extracted after exacerbation of symptoms
Fawzy et al. (2020)	1	N/S	Temporary filling and occlusal adjustment	Glass‐ionomer restoration of the access cavity	1 year	The tooth survived and there was radiographic healing of the apical lesion
Gutmann and Rakusin (1994)	2	N/S	Orthodontic bands and occlusal adjustment	Glass‐ionomer core build‐up with intra‐orifice barriers and crowns	8–16 months	Both teeth survived without symptoms
Ito et al. (1998)^±^	1	See Table 2	N/S	Crown	2 years	Tooth survived
Jun et al. (2019)	1	N/S	N/S	Crown	3 years	Tooth survived
Liu and Sidhu (1995)	6	Cracks removed to determine their extent	Orthodontic bands (occlusal splint was also provided for one of the cases)	Intra‐radicular amalgam cores and crowns (two of the cases had not received a definitive restoration at review)	1–3.5 years	All cases survived
Mahgoli et al. (2019)	4	Crack lines were disinfected with chlorhexidine and sealed with a self‐cure resin cement	Occlusal adjustment	Post‐and‐core build‐ups and crowns	1.5–10 years	All cases survived
Michaelson (2015)	3	Complete removal of crack lines with a surgical bur or an ultrasonic tip and repair of the iatrogenic perforation with mineral trioxide aggregate	N/S	Crack excision and perforation repair were performed through existing crowns	1–2 years	All cases survived and remained asymptomatic
Michaelson (2017)	3 – same cases with Michaelson (2015)	Same as Michaelson (2015)	N/S	Same as Michaelson (2015)	3.5–5.5 years	All cases survived and remained asymptomatic
Ritchey et al. (1957)^±^	1	N/S	Temporary crown	Crown	20 months	Tooth survived

*Note*: Olivieri et al., mention regarding the treatment of cracked teeth with normal pulp or reversible pulpitis.

● §: Based on data obtained through personal communication by Olivieri et al. (2020).

**Table 3:** Clinical studies with mixed/unspecified pulpal diagnoses and treatments

Study	No of teeth	Treatment	Follow‐up	Outcomes
**Observational studies (*n*** **=** **9)**				
Abou‐Rass (1983)	120	Crowns with/without endodontic treatment	Not specified, up to 9 years	86% tooth survival
Brynjulfsen et al. (2002)	46	Direct or indirect cuspal coverage (50%), endodontic treatment (about 50%), extraction (less than 5%)	2 years	About 95% tooth survival, 90% symptom free
Cameron (1976)	102	Crowns (50%), onlays (25%), endodontic treatment or extraction (25%)	Not specified, up to 10 years	All initially vital teeth (75%) remained vital, all endodontically treated teeth were preserved
Ferracane et al. (2022)	2858	Monitoring (64%), crowns (22%), direct or indirect partial restorations (13%), endodontic treatment (less than 1%), splints or desensitizing (less than 0,01%)	3 years	Tooth survival over 98%; 80% of teeth initially recommended for monitoring progressed with a monitoring recommendation
Hilton et al. (2020a)	1850	Monitoring	1 year	23% of teeth experienced a decrease in baseline symptoms, 10% an increase
Hilton et al. (2020b)	2858	Same as Ferracane et al. (2022)	Not specified	Not specified, preliminary report of Hilton et al. (2020a) and Ferracane et al. (2022)
Lee et al. (2021b)	68 (2009 cohort), 184 (2019 cohort)	Provisional crown + definitive crown (131 teeth), provisional crown + endodontic treatment before (104) or after (17) definitive crown	11 years (2009 cohort), 1 year (2019 cohort)	Tooth survival of 95% (2009 cohort) and 100% (2019 cohort)
Liao et al. (2022)	77	Monitoring (27%), Direct composite (1.3%), endodontic treatment + crown (23%), provisional crown + definitive crown without (10%) or with (7%) endodontic treatment, orthodontic band + crown without (2.6%) or with (18%) endodontic treatment, extraction (9%)	2 years	62.8% overall tooth survival (recall rate was 45%), 81% for monitoring or direct composite, 76% with crowns
Roh and Lee (2006)	154	Crowns without (42%) or with (43%) endodontic treatment, direct composite (2%), extraction (13%)	Not specified, up to 1 year	87% tooth survival, 44% pulp survival

## 3 MONITORING VERSUS RESTORING

Do all teeth diagnosed with cracks require some form of treatment? A practice‐based observational study revealed that only about one‐third of 2858 vital cracked teeth were recommended for restoration (Hilton et al., 2020b). Presence of caries, pain on biting, radiographic evidence of a crack, and spontaneous pain were the strongest predictors towards proceeding to restoration. In contrast, teeth with exposed roots were more likely advised for monitoring, potentially due to the fact that symptoms on such cases were attributed to dentine hypersensitivity.

Besides, symptoms remained unchanged after 1 year in more than two thirds of 1850 untreated teeth from the above cohort (Hilton et al., 2020a). Interestingly, reductions in symptoms, especially pain to cold, were over twice as common as increases (23% and 10%, respectively) and there was a greater trend toward decreasing symptoms in patients that had been initially recommended for treatment, but had not been performed (45%), compared to patients that had initially been advised for monitoring (19%). Female gender, molar teeth, crack involving the distal or buccal surface and parafunction were independently associated with a decrease in symptoms, whilst the presence of mesial crack was associated with an increase. Moreover, about 80% of teeth among those initially recommended for monitoring progressed with a monitoring recommendation over a period of 3 years (Ferracane et al., 2022).

Monitoring was also implemented by Liao et al. (2022) for 21 teeth, in which crack lines were barely visible or incipient. Despite the low recall rate of nearly 50%, it was described that about 80% of the teeth remained asymptomatic after 2 years. Furthermore, Kanamaru et al. (2017) monitored two teeth with cracks extending to the middle and deep part of dentine respectively, which both remained vital over 1‐3 years of follow‐up. However, it is not clear how crack extension was determined without any intervention.

### 3.1 Concluding remarks

Current evidence suggests that rapid intervention is not always needed for cracked teeth, as the progression of symptoms is slow and may even be directed towards the opposite side from what is generally expected. Nonetheless, the above findings should be interpreted with caution. The included samples were not randomly recruited, thus the outcomes could be potentially influenced by the presence of confounding factors, such as the severity of baseline symptoms. In addition, given the short‐term observation periods, the key questions are for how long an untreated cracked tooth can remain stable and whether prompt intervention is preferrable to avoid future complications. These remain to be addressed with well‐designed controlled studies in the future.

## 4 MANAGEMENT OF CRACK LINES AND ASSOCIATED PERIODONTAL POCKETS

When a cracked tooth has been diagnosed and decision has been taken to embark on treatment, clinicians often face the dilemma of whether to remove crack lines or not.

### 4.1 Rationale for crack removal

With regard to diagnostic aspects, tracing crack lines may be valuable so as to determine their exact location and extent as well as to evaluate pulp vitality in ambiguous cases (when performed without local anesthesia) (Abou‐Rass, 1983) to decide the next stage of approach. For example, endodontic treatment was performed when tracing of the crack line revealed direct communication with the pulp (Liu & Sidhu, 1995) or even extraction when investigation of the crack disclosed extension to the pulpal floor (Ritchey et al., 1957).

In terms of biological aspects, it has been confirmed that crack lines are colonized with bacteria, arranged in biofilms, which invade dentinal tubules along with their by‐products and induce pulpal inflammation (Ricucci et al., 2015). This was corroborated by red fluorescence emission that was observed through the crack lines and has been indicative of porphyrin, a by‐product of bacterial metabolism (Jun et al., 2019). Nevertheless, intratubular bacterial ingress can be dependent on numerous variables, such as crack direction (Ricucci et al., 2015), hydrostatic pressure changes during mastication (Michelich et al., 1980) and defensive mechanisms of the pulp (Pashley, 1996) while pulpal response to the bacterial challenge can also vary according to the crack extent (Ricucci et al., 2015), the concentration and relative virulence of bacterial by‐products, the infected area of dentine and the pulpal state (Pashley, 1990).

### 4.2 Crack line management

#### 4.2.1 Teeth with normal pulp (NP) or reversible pulpitis (RP)

Regarding cracked teeth with NP/RP, some researchers preferred to eliminate crack lines completely and proposed the use of fiber‐optic transillumination so as to confirm complete crack removal (Abbott & Leow, 2009; Batalha‐Silva et al., 2014). Although only two out of the 100 teeth in the study by Abbott and Leow (2009) required endodontic treatment due to pulp exposure during complete crack removal, other authors claimed that this approach increases the risk of iatrogenic pulp damage (Griffin, 2006) and preferred to partially remove crack lines, especially in the absence of related pocket depths that denote deeper crack extension (Ito et al., 1998). As for the endpoints of partial crack removal, Liebenberg (1996) traced crack lines until they diminished to a fine craze, Griffin (2006) removed cracks until no separation of the tooth could be felt with a sharp probe while Lee et al. (2021a) terminated the crack removal procedure when a shallow crack remained close to the pulp and lined the crack with flowable composite.

In contrast, other research teams preferred to leave crack lines in situ and provided full coverage restorations to splint the fractured elements of the tooth and prevent further crack progression (de Toubes et al., 2020; Krell & Rivera, 2007). Specifically, in the former study previous restorations were left in place before providing crowns while in the latter, restorations were removed, and crack lines were disinfected with chlorhexidine. However, a finite element analysis on three‐dimensional cracked tooth models showed that resistance to crack propagation obtained with a crown restoration was significantly higher when this was combined with crack removal and a composite core build‐up (Kim et al., 2021). In addition, 21% of the teeth in the study by Krell and Rivera (2007) required endodontic treatment within 6 months of the crown placement, implying that mechanical splinting only may not be sufficient when cracks remain.

#### 4.2.2 Teeth with pulpal involvement

In a case series with previously endodontically treated teeth having cracks involving the pulpal floor, crack line was sealed with a self‐cure resin cement after being disinfected with chlorhexidine (Mahgoli et al., 2019). The authors claimed that cement selection was based on its similarity of elasticity to that of dentine, which would result in superior stress distribution and prevention of stress concentration at the crack site. Likewise, in cases of cracks extending beyond the canal orifices, flowable resin was applied with a size 6 K file under microscope magnification to seal the crack line (Malentacca et al., 2021). Another more radical approach regarding radicular cracks included complete removal of the crack line with a surgical bur or an ultrasonic tip and repair of the iatrogenic perforation with mineral trioxide aggregate (Michaelson, 2015; Michaelson, 2017). Although a perforation cannot be considered as an ideal clinical scenario, the research team reported that when it is performed under controlled circumstances (minimal size and immediate repair), it could result in long‐term clinical success (all 3 cases remained asymptomatic and with improved periodontal status after 3.5–5.5 years). In contrast, other investigators, who focused on cracks being mainly a mechanical rather than a biological complication, did not make any effort to eliminate the crack lines (Tan et al., 2006). Instead, they provided coronal protection with crowns or orthodontic bands to immobilize the cracked segments and prevent further crack propagation.

### 4.3 Management of associated periodontal pockets

Periodontal implications can derive from bacterial leaching through the crack line in cracked teeth experiencing pulpal involvement (Gutmann & Rakusin, 1994), thus certain investigators incorporated periodontal intervention into their treatment protocol. After completion of restorative procedures, Malentacca et al. (2021) proceeded to thorough polishing of the crack line inside the periodontal pocket to prevent any further bacterial entrapment and they also rinsed the pocket with chlorhexidine solution. This was followed by sulcular placement of chlorhexidine gel, which was repeated four times in 5‐day intervals. Similarly, in a case series that iatrogenic perforation was attempted to completely eliminate the crack (Michaelson, 2015), periodontal healing was enhanced by a single application of a local antibiotic agent one month thereafter.

### 4.4 Concluding remarks

It is not possible to provide solid recommendations as for the management of crack lines and associated periodontal pockets. This is attributed to the available data deriving from observational studies, case reports and in vitro investigations, thus having a high potential of bias in their findings. Additionally, the majority of cracked tooth studies did not report their protocol for management of cracks or pockets. Therefore, future controlled studies, ideally randomized, should be designed to reliably assess the effect of these approaches on the outcomes of cracked teeth.

## 5 RESTORATIVE APPROACHES FOR CRACKED TEETH WITH NP/RP

There are two main trends in the literature for restoring cracked teeth with baseline diagnosis of NP/RP. These can be classified into ‘single‐stage treatment’ and ‘multiple‐stage treatment’. For the purposes of this review, these terms can be defined as follows:

- Single‐stage treatment: immediate provision of a definitive type of restoration, either direct or indirect, following diagnosis of cracked tooth with NP/RP.
- Multiple‐stage treatment: a staged approach where definitive restoration of a cracked tooth with NP/RP is provided after an interim treatment and review of the progression of symptoms.

### 5.1 Single‐stage treatment

This approach has been described with direct, indirect partial‐coverage, and full‐coverage restorations (Table 1).

#### 5.1.1 Direct restorations

Direct resin composite or amalgam restorations, with and without cuspal coverage, have been used as a single‐stage treatment for cracked teeth with NP/RP after removal of previous restorations.

Regarding amalgam, Davis and Overton (2000), who randomly allocated 40 patients to bonded and pin‐retained restorations, reported elimination of bite pain for both groups after 2 weeks, whereas cold sensitivity was eliminated only for the bonded group at 3 months and remained unchanged in the mechanical group up to the 12‐month review. Bonded amalgam was also successful in eliminating symptoms of bite pain and cold sensitivity in four case reports with follow‐ups ranging from 15 to 26 months, where previous mechanically retained amalgam restorations had been replaced (Bearn et al., 1994). On the other hand, Homewood (1998) demonstrated that mechanically retained amalgam could alleviate symptoms in nearly 94% of 48 cracked teeth after 15 months; however, the most common symptom in that cohort was biting pain with less than half of teeth showing cold sensitivity.

**Figure 2:**
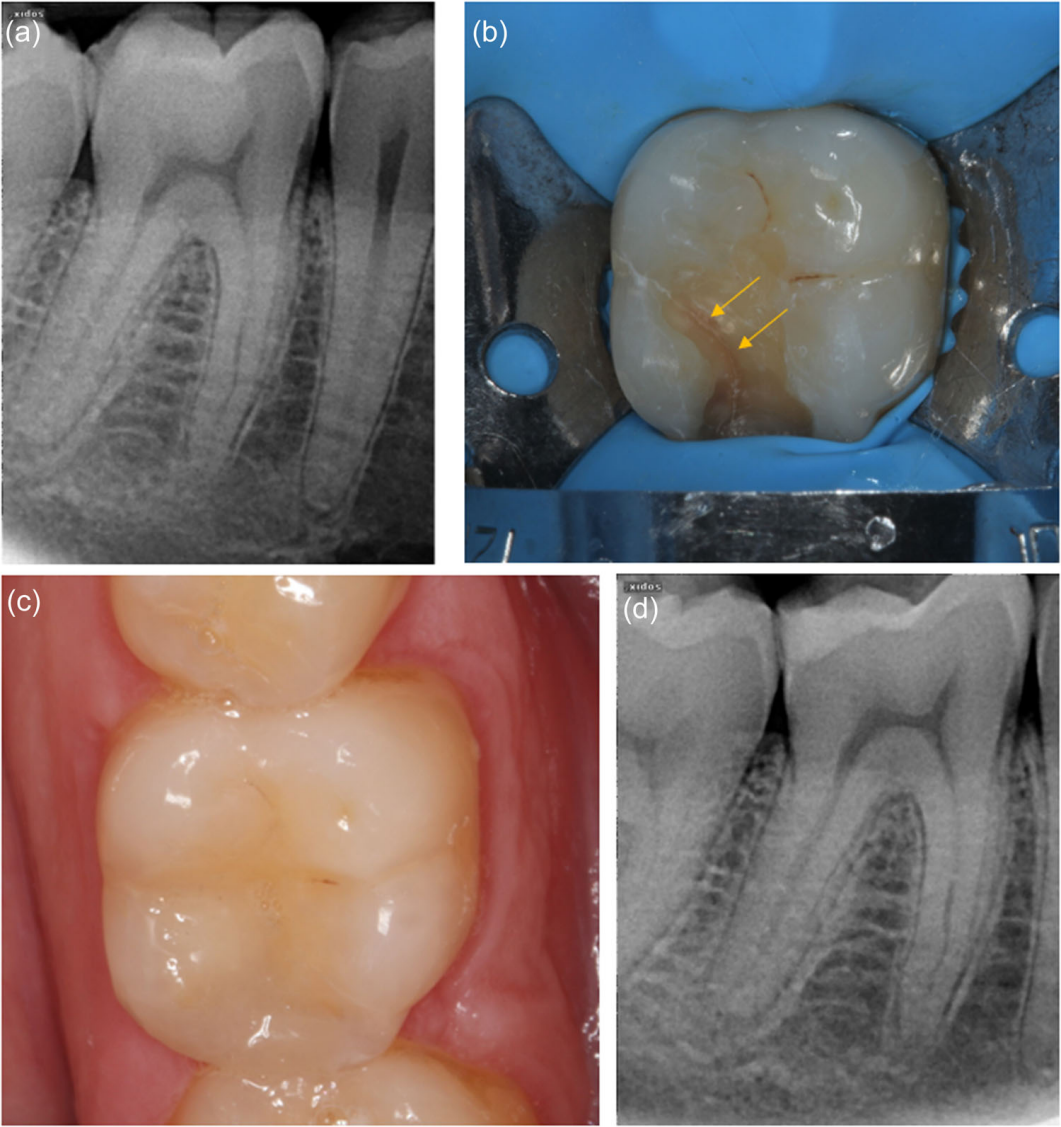
Direct composite restoration in an intact cracked 46 with reversible pulpitis (a) preoperative long‐cone periapical radiograph (b) crack line visible (arrows) after investigation with bur (c) composite restoration completed (d) periapical radiograph at 6 months follow‐up; the tooth has remained vital and asymptomatic.

More long‐term data are available for direct composite (Figure 2). These mainly derive from Opdam et al. (2008), who investigated 40 cracked teeth with RP and bite pain. Despite only half of the teeth being symptom‐free at the 6‐month review, 37 teeth (93%) remained vital after an observation period of 7 years. Among them, 30 teeth were completely asymptomatic while seven were more sensitive than the adjacent controls to cold testing. Two of the three teeth that underwent endodontic treatment, were finally extracted or hemisected due to developing vertical root fracture. The same study also demonstrated non‐significant effect of cuspal coverage in terms of pulp or tooth survival, after randomly allocating teeth for direct restorations with and without cuspal coverage (Opdam et al., 2008). Nevertheless, cuspal coverage restorations were significantly more effective in terms of restoration failures; no failures were reported for cuspal coverage direct composite over 7 years while restorations without cuspal coverage had a mean annual failure rate of 6%, although failures were repairable (fracture, secondary caries, and wear).

In addition, nonsignificant effect of cuspal coverage was reported in the in vitro fatigue resistance of mesial‐occlusal‐distal direct composite restorations in extracted third molars with simulated crack lines (Naka et al., 2018). It is evident though that laboratory results cannot truly replicate clinical conditions. For example, only vertical loading was tested in this study, whereas lateral forces seem to play a crucial role in the mechanism of cracked teeth clinically (de Toubes et al., 2022; Kanamaru et al., 2017).

#### 5.1.2 Indirect partial‐coverage restorations

**Figure 3:**
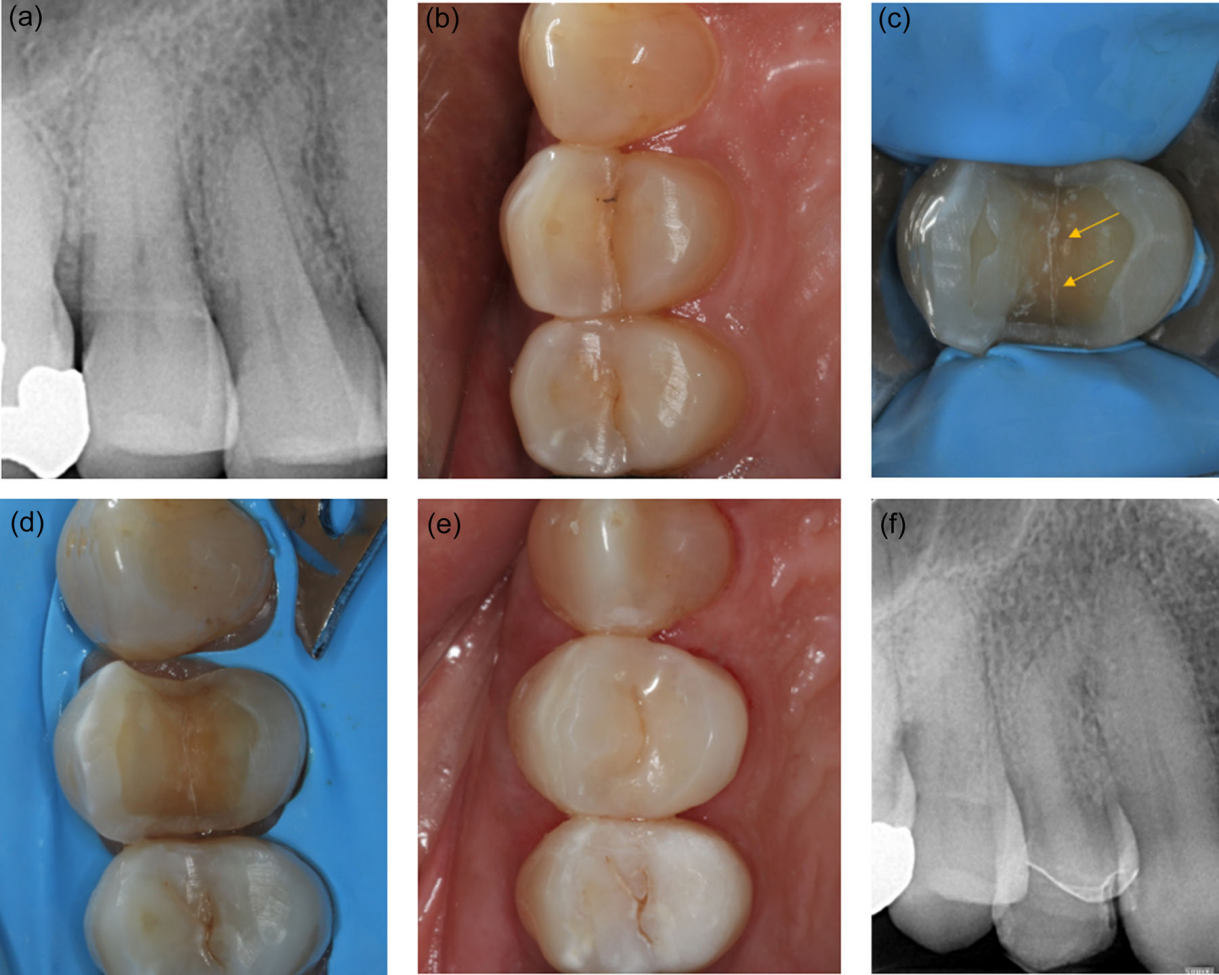
Indirect composite onlay restoration in an intact cracked 14 with reversible pulpitis (a and b) preoperative long‐cone periapical radiograph and occlusal view (c) crack line revealed (arrows) after investigation with bur (d) onlay preparation (e) postoperative occlusal view with bonded indirect composite onlay (f) periapical radiograph at 6 months follow‐up; the tooth has remained vital and asymptomatic.

Indirect partial restorations with and without cuspal coverage (onlays and inlays respectively) have also been employed as a single‐stage treatment for cracked teeth with NP/RP (Figure 3). In vitro data demonstrated higher fatigue resistance of indirect composite onlays compared to inlays for molars with simulated cracks (Magne et al., 2012) (Table 4). Clinically, the decision between indirect inlays and onlays has been based on the criterion of pain under cuspally induced flexure (Liebenberg, 1996). Inlays have been advocated for teeth in which sensitivity is not exacerbated by chewing or bite testing devices (Kang et al., 2016), whilst cuspal coverage has been generally preferred for teeth that are positive to bite testing (Griffin, 2006; Liebenberg, 1996; Signore et al., 2007).

**Table 4:** In vitro studies related to the treatment of cracked teeth

Study	Specimens	Method	Restorations	Outcomes
**Studies relevant to teeth with normal pulp or reversible pulpitis (*n*** **=** **3)**				
Kim et al. (2021)	Three‐dimensional cracked tooth models	Finite element analysis	Direct/indirect composite, ceramic inlays/onlays, gold crown with/without resin core	Ceramic inlays/onlays and gold crown with resin core showed the most favorable stress distribution
Magne et al. (2012)	Extracted human third molars with standardized MOD^+^ cavities and simulated cracks	Cyclic fatigue test	Indirect composite inlays/onlays with/without fiber patch	Significantly higher fatigue resistance of onlays compared to inlays; no effect of fiber patch
Naka et al. (2018)	Extracted human third molars with standardized MOD^+^ cavities and simulated cracks	Cyclic fatigue test	Direct/indirect composite with/without cuspal coverage	Significantly higher fatigue resistance of direct groups compared to indirect; no significant effect of cuspal coverage
**Studies relevant to endodontically treated teeth (*n*** = **3)**				
Anton Y. Otero et al. (2021)	Extracted human third molars with simulated cracks and endodontic treatment	Cyclic fatigue test	CAD/CAM^±^ resin composite endocrowns with/without fiber‐reinforced composite base	A fiber‐reinforced base did not significantly affect the fracture resistance of teeth restored with endocrowns, but it resulted in more fractures being restorable (50‐80%) in comparison to the control group (30%)
Lin et al. (2013)	Three‐dimensional finite element premolar models with different crack depths	Finite element analysis	CAD/CAM^±^ ceramic crowns or onlays or endocrowns	Onlays exhibited higher failure probabilities compared to both crowns and endocrowns; endocrowns provided comparable fracture resistance to crowns in cases of shallow cracks (about 1 mm above bone level), but they showed higher failure risk for deep cracks (below bone level to mid‐root)
Shi et al. (2021)	Three‐dimensional finite element models with simulated cracks and endodontic treatment	Finite element analysis and fracture failure test	Crowns and onlays with/without different types of fiber reinforcement	Crowns resulted in superior fracture resistance than onlays when both combined with an annular fiber‐reinforced base; fiber‐reinforced onlays exhibited significantly more favorable fracture pattern compared to crowns

Signore et al. (2007) performed 43 indirect composite onlays in cracked teeth with bite pain and cold sensitivity and reported that 93% of cases remained vital and asymptomatic after 6 years. These results are comparable with those of direct composite restorations, as shown by Opdam et al. (2008), given that both studies had similar sample size, follow‐up time, baseline symptoms, and type of pre‐existing restorations (amalgam). However, remission of symptoms was more rapid with indirect restorations, with 88% of teeth being symptom‐free after only a week (Signore et al., 2007). Possible explanations involve the subjectivity in the evaluation of symptoms and the fact that cuspal coverage was employed in all cases investigated by Signore et al. (2007), in comparison to only half of the cases reported by Opdam et al. (2008). On the other hand, indirect restorations have been associated with inferior fracture resistance compared to direct (Naka et al., 2018) while the use of a polyethylene fiber patch as a core reinforcement offered no additional benefit (Magne et al., 2012) (Table 4). Failures for indirect groups were mainly cohesive, with the lack of adhesive failures possibly attributed to immediate dentine sealing (Magne et al., 2012; Naka et al., 2018).

Another drawback of indirect compared to direct restorations is considered to be the need for provisionalization, which may increase the risk of pulpal complications and cuspal deflection in cracked teeth (Griffin, 2006; Guthrie & DiFiore, 1991). A method to overcome this includes the provision of indirect restorations at a single visit via chairside computer‐aided‐design/computer‐assisted‐manufacturing (CAD/CAM) systems (Griffin, 2006). Another option, in accordance with the principles of immediate dentine sealing, is to provide a direct composite pre‐reconstruction immediately after removing previous restorations and before the impression for the definitive restoration (Signore et al., 2007). This approach, apart from preventing cuspal flexure, may facilitate a uniform depth of tooth preparation (Signore et al., 2007) and increase the bond strength of the definitive restoration (Magne et al., 2012; Naka et al., 2018).

As for material selection, ceramic indirect partial coverage restorations were shown to be superior to direct or indirect composite in preventing further crack propagation in cracked teeth, even though composite exhibited higher stress absorbing capacity (Kim et al., 2021). This was associated with the increased modulus of elasticity of ceramic that prevented separation of the cracked segments despite the higher stress concentration at the restoration‐tooth interface. Nevertheless, clinical data for ceramic (Griffin, 2006; Liebenberg, 1996) or metal onlays (Chana et al., 2000; Marchan et al., 2013; Yap, 1995) derive solely from a limited number of case reports or case series with small sample sizes (up to six teeth), thus further clinical investigation is needed.

#### 5.1.3 Full crowns

A crown has been reported to be the predominant type of restoration for cracked teeth with NP/RP (approximately twothirds of total restorations) (Hilton et al., 2020b). Wu et al. (2019) claimed that a crown could be more effective than other types of cuspal coverage restorations in encompassing deep cracks at the corono‐apical dimension while crown preparation may contribute to the removal of crack lines. Given the importance of an enamel peripheral rim for the predictability of bonded restorations, a crown has been considered preferrable when cracks extend below the cementoenamel junction and could not diminish into a fine craze within enamel limits (Liebenberg, 1996). Pocketing associated with deep crack extension (Marchan et al., 2013) and the presence of occlusal interferences (Kanamaru et al., 2017) have also been suggested as indications for a crown.

However, as previously mentioned, in vitro fracture resistance obtained with a crown restoration, cemented with resin‐modified glass ionomer cement, was influenced by previous crack removal and a composite core build‐up (Kim et al., 2021) (Table 4). When the crown was combined with crack removal and a composite core, resistance to crack propagation was higher compared to direct or indirect composite and comparable to ceramic indirect partial coverage restorations. On the contrary, when provided without crack removal and a resin core underneath, stress concentration at the restoration‐crack interface as well as the crack margins was the highest among the aforementioned groups of restorations.

In terms of clinical data, Krell and Rivera (2007) provided crowns as a single‐stage treatment for 127 cracked teeth with RP. 27 of these teeth (21%) required endodontic treatment due to irreversible pulpitis or pulp necrosis within 2 and 5 months from the provision of crowns respectively. Within the limitations of indirectly comparing findings from different studies, it can be highlighted that this percentage of pulpal complications is considerably higher compared to the respective one reported for direct or indirect composite restorations (7%) (Opdam et al., 2008; Signore et al., 2007) and slightly higher than crowned teeth in general (15%–19%) (Cheung et al., 2005; Saunders & Saunders, 1998). Possible reasons for the impact on pulpal health could involve the damage caused by the substantial amount of tooth preparation that is required for a crown and the treatment protocol used in that study, as neither previous restorations and cracks were removed nor a bonded core was performed.

### 5.2 Multiple‐stage treatment

As mentioned previously, a multiple‐stage approach includes provision of an interim treatment so as to monitor pulpal condition and confirm its reversible state before recommending a definitive restoration (Ehrmann & Tyas, 1990). Interim treatments can be classified into the following categories:

- Extra‐coronal splinting.
- Intracoronal restorations.
- Bidirectional splinting.
- Adjunctive methods.

The following sections will discuss the indications, proposed duration and types of interim treatment, as well as the available data on the effect of different multiple‐stage treatment approaches on pulp and tooth survival (Table 1).

#### 5.2.1 Indications and duration of interim treatment

An interim treatment was recommended when more prolonged cold sensitivity, signifying potential stimulation of C fibers, was recorded (Homewood, 1998; Kang et al., 2016) as well as in the presence of bite pain (Kang et al., 2016). However, the threshold of the duration of pain after stimuli that are considered indicative of RP was not specified by the above studies, while a considerable variability has generally been recorded in the literature of cracked teeth, with a range of 5 s (Krell & Rivera, 2007) to 45 s (Davis & Overton, 2000). In addition, the sensitivity of bite tests may be influenced by the method used, as cotton rolls provided less accuracy than dedicated bite blocks (Yang et al., 2019). These variations could explain contradictory approaches; for example, single‐stage treatment has produced satisfactory results for teeth with bite pain, as previously mentioned (Opdam et al., 2008; Signore et al., 2007).

The restorative status was another factor taken into consideration due to the hypothesis that cracks may be more superficial in heavily restored teeth compared to intact or minimally restored teeth since crack propagation was expected to follow a direction parallel to the cuspal incline (Homewood, 1998) with stresses concentrating at the restoration‐tooth interface (Roh & Lee, 2006). In contrast, no difference has been reported between restored and unrestored cracked teeth in terms of pulp preservation (Kanamaru et al., 2017; Lee et al., 2021a).

Regarding the duration of interim treatment, a considerable discrepancy has been exhibited in the literature, with observation periods ranging from 1 week (de Toubes et al., 2020; de Toubes et al., 2022) to 6 months (Ito et al., 1998), whilst many studies did not report the follow‐up of their interim treatment (Kang et al., 2016; Kim et al., 2013; Roh & Lee, 2006).

Advocates of longer review periods underlined the importance of allowing ample time for the pulp to heal to confirm the initial diagnosis of RP (Abbott & Leow, 2009; Wu et al., 2019). It has been demonstrated that the pulp needs a period of 4–8 weeks to recover after an episode of bacterial insult (Bergenholtz et al., 1982; Warfvinge & Bergenholtz, 1986). Thus, restorative procedures, such as crown preparation, at a shorter follow‐up time might further compromise the status of the inflamed pulp (Wu et al., 2019). Allowing a longer period could also facilitate distinguishing a normal pulp from an asymptomatic necrotic pulp, given the subjective nature of sensibility tests (Abbott & Leow, 2009).

On the other hand, authors in favor of short‐term interim treatment or single‐stage treatment claimed that most types of restorations used as interim treatments are not effective in preventing crack propagation while also exhibiting increased risk of inducing further pulpal inflammation due to microleakage or dislodgement during the interim treatment period (de Toubes et al., 2022; Guthrie & DiFiore, 1991; Wu et al., 2019).

#### 5.2.2 Extra‐coronal splinting

The rationale of extra‐coronal splinting is to immobilize the cracked segments to relieve symptoms from their independent movements upon application of masticatory forces as well as to prevent further crack propagation (Guthrie & DiFiore, 1991). Interim treatments based on extra‐coronal splinting include stainless steel orthodontic bands, temporary crowns, and the supra‐coronal direct composite splint.

Interim treatment with stainless steel orthodontic bands was first described by Ehrmann and Tyas (1990) for three teeth that had remission of symptoms after 2–4 weeks and were definitely restored with crowns. Subsequent studies using this protocol for 1–3 months showed cessation of RP and bite pain symptoms in 83%–100% of cases (Homewood, 1998; Wu et al., 2019). However, prolonged treatment with orthodontic bands has been associated with an increased risk of pulpal complications; 5‐year estimated pulp vitality rate was 81% after definitive crown placement and 37% when orthodontic bands remained due to patients refusing definitive treatment with crowns (Wu et al., 2019). This difference was attributed to the lack of customized fitting and occlusal coverage of orthodontic bands, as well as the higher risk of cement breakdown due to its exposure to the oral environment.

Provisional crowns were introduced as an interim treatment option by Guthrie and DiFiore (1991), who claimed that the occlusal coverage, as well as the retention and resistance form of crowns, could provide more effective protection from masticatory forces compared to orthodontic bands. In their study, 89% of cases remained vital and asymptomatic after 2 weeks of observation with provisional crowns and subsequently, received definitive crowns. The lower pulp survival rates (58%–71%) after provisional crown placement that were described by two university hospital‐based retrospective studies (Kang et al., 2016; Kim et al., 2013) were attributed to the delayed referral of patients.

Another form of extracoronal splinting, the supra‐coronal direct composite splint (DCS), involves placement of a bonded resin composite restoration in supra‐occlusion, without any tooth preparation, encompassing the entire occlusal surface as well as the occlusal third of the axial surfaces of the cracked tooth (Banerji et al., 2014). According to these authors, DCS may overcome the periodontal and esthetic shortcomings of orthodontic bands as well as the biologically invasive nature of temporary crowns by being reversible and also having the potential to provide interocclusal clearance for definitive restorations without tooth tissue removal. Their retrospective study showed remission of RP and bite pain in 86.6% of the overall 151 cases after an evaluation period of 3 months while more than 97% of these had re‐establishment of occlusal contacts (Banerji et al., 2014). Failures included the development of irreversible pulpitis for 11 cases (7%), restoration fractures or debonding (five cases), and intolerance of the supra‐coronal restoration (four cases). Nevertheless, true intolerance rates are likely to be higher as the included sample had been previously tolerant to a trial (unbonded) splint. In addition, cases potentially unsuitable for supra‐coronal restorations (reduced eruptive potential, unstable periodontitis, temporomandibular disorders, previous orthodontic treatment) were excluded.

#### 5.2.3 Direct intra‐coronal interim restorations

Direct intra‐coronal restorations, either with glass‐ionomer (Abbott & Leow, 2009) or composite (de Toubes et al., 2020; de Toubes et al., 2022; Ito et al., 1998), have also been suggested as interim treatments for cracked teeth with NP/RP. The relevant studies agreed in terms of removing all previous restorations, whereas complete or partial removal of crack lines was reported only by Abbott and Leow (2009) and Ito et al. (1998), respectively. Besides, the duration of these interim treatments ranged from 1 week (de Toubes et al., 2020; de Toubes et al., 2022) to 3 months (Abbott & Leow, 2009) and 6 months (Ito et al., 1998).

With regard to findings from observational studies, the approach involving crack removal and a longer duration of interim treatment (Abbott & Leow, 2009) resulted in lower pulp survival rate after the interim treatment (80%) compared to the study by de Toubes et al. (2022) (100%) but prevented pulpal complications following definitive restoration (0% vs. 12%, respectively). It should also be highlighted that the former study included a larger sample (100 vs. 26 teeth), although with a low recall rate (54%).

#### 5.2.4 Bidirectional splinting

Bidirectional splinting consists of a combination of extra‐coronal splinting and an intra‐coronal direct restoration. This stepwise approach includes a relatively short‐term course of orthodontic band (up to 3 weeks) followed by crack removal and direct intra‐coronal restoration (Batalha‐Silva et al., 2014; Lee et al., 2021a). A further 1‐month review of a temporary crown was employed in the prospective study by Lee et al. (2021a) before providing the definitive crown restoration. Pulp vitality in this study was preserved for 72% of cases after the interim treatment period, however, pulp survival rate after definitive crown placement was 91%, and no tooth was lost after a mean follow‐up period of 2.6 years.

#### 5.2.5 Adjunctive methods

Additional approaches, such as occlusal adjustment and the use of sedative liners, have been employed mainly as adjuncts to the above interim treatments.

Occlusal adjustment has been used along with intracoronal direct restorations and DCS, either by reduction of the cracked tooth (de Toubes et al., 2020, 2022) or by performing composite additions in guiding teeth so as to prevent excursive contacts on the cracked tooth (Banerji et al., 2014; Ito et al., 1998). Banerji et al. (2014) found no significant effect of this adjustment on the failure rate of DCS, while no conclusions can be derived from the other studies due to their limited sample size (de Toubes et al., 2020; Ito et al., 1998) or insufficient data (de Toubes et al., 2022). When provided as a sole interim treatment in 25 vital cracked teeth linked to occlusal interferences, occlusal adjustment demonstrated limited benefit in preventing pulpal complications, as nearly half of the teeth finally underwent endodontic treatment (Kanamaru et al., 2017). A possible explanation for this low effect may be that overloading of the tooth could still occur after contact with a food bolus (Hiatt, 1973).

With regard to liners, various types including zinc‐oxide eugenol (Ehrmann & Tyas, 1990; Ritchey et al., 1957), an antibiotic‐corticosteroid compound (Abbott & Leow, 2009), calcium hydroxide (de Toubes et al., 2020) and glass‐ionomer cement (de Toubes et al., 2022), have been used to sedate the inflamed pulp. A retrospective study that reported eugenol sedation as a sole interim treatment for 9 teeth, showed that endodontic treatment was required for a third of the sample (Kanamaru et al., 2017). Similarly, zinc‐oxide eugenol failed to preserve pulp vitality in an earlier case report (Ritchey et al., 1957). Generally, the efficacy of liners is difficult to be assessed as these were predominantly combined with other forms of interim treatments, such as orthodontic bands (Ehrmann & Tyas, 1990) or direct intra‐coronal restorations (Abbott & Leow, 2009; de Toubes et al., 2020, 2022).

#### 5.2.6 Definitive restorations

It should be recognized that the evidence regarding the performance of definitive restorations in multiple‐stage approaches is compromised since many studies limited their follow‐up to the interim treatment period (Banerji et al., 2014; Kang et al., 2016; Kim et al., 2013) or included mixed cohorts with teeth that required endodontic treatment from baseline (Lee et al., 2021b; Liao et al., 2022) (Table 3) or did not specify the number of cases that were assigned to different types of definitive restorations (Abbott & Leow, 2009; Homewood, 1998).

Within these limitations, it can be noted that the majority of relevant investigations selected crowns as definitive restorations with alternatives including indirect onlays or direct composite restorations (Table 1). Studies that exclusively used crowns reported 81%–100% pulp survival rates 1–3 years after final restoration (Guthrie & DiFiore, 1991; Lee et al., 2021a; Wu et al., 2019) while similar performance (83%–100% after 15 months to 5 years) was shown by studies that used either crowns or indirect onlays (Abbott & Leow, 2009; de Toubes et al., 2022; Homewood, 1998).

It may be highlighted that the lowest pulp outcomes derived from the studies that used orthodontic bands as interim treatments (81%–83%) (Homewood, 1998; Wu et al., 2019). When other types of interim treatments were employed, the rates of pulp complications after definitive restoration (0%–12% after up to 5 years) were lower than those reported by Krell and Rivera (2007) (21% after 6 months), who performed crowns as single‐stage treatment, and comparable to the pulp outcomes reported for single‐stage direct (Opdam et al., 2008) and indirect (Signore et al., 2007) composite restorations (7% after 6–7 years). This could indicate that the stepwise approach of multiple‐stage treatment may limit the risk of pulpal complications after provision of the definitive restoration, at least regarding crowns. This is important as endodontic treatment in such occasion would lead to removal or deterioration of the final restoration (Lee et al., 2021a).

In terms of tooth survival, overall high rates (96‐100%) were reported after up to 5 years of observation regardless of the type of interim or definitive treatment used (Abbott & Leow, 2009; de Toubes et al., 2022; Homewood, 1998; Lee et al., 2021a; Wu et al., 2019).

### 5.3 Concluding remarks

Given the paucity of controlled studies and the susceptibility to bias of the surrogate findings of observational research, it is not possible to derive tangible conclusions regarding the restorative approaches for cracked teeth with NP/RP. Within these shortcomings, the followings points could be highlighted:

- As for single‐stage treatment, current clinical evidence supports direct and indirect composite restorations, which have been associated with high pulp survival rates (93%) over 6–7 years of follow‐up. There is a weak indication of cuspal coverage being advantageous, especially regarding restoration failures.
- When a crown is preferred as definitive restoration, multiple‐stage treatment has been linked to reduced posttreatment pulpal complications compared to the single‐stage approach.

- The ideal duration of an interim treatment remains contentious. Long‐term presence may not be indicated for some methods (orthodontic bands) whereas it could be beneficial for others (re‐establishment of occlusal contacts with DCS).

- Regardless of the single or multiple‐stage approach, the available data suggest high rates of tooth survival (95%–100%) over 5–7 years.

Future studies, ideally in the form of randomized controlled trials, should aim to perform comparisons between single and multiple‐stage approaches as well as within each approach as for their effect on pulp and tooth survival.

## 6 ENDODONTIC TREATMENT IN CRACKED TEETH

### 6.1 Indications for endodontic treatment

When does a cracked tooth require endodontic intervention? The various reasons that have been reported in the literature could be categorized as follows:

- Diagnosis of pulpal pathoses (irreversible pulpitis or pulp necrosis) either at baseline (Davis & Shariff, 2019; Kang et al., 2016; Liao et al., 2022; Lu et al., 2021) or after initial management of cracked teeth that presented with baseline NP/RP (de Toubes et al., 2022; Krell & Rivera, 2007; Lee et al., 2021a; Opdam et al., 2008). However, conflicting approaches considered teeth that resulted in pulp necrosis due to cracks as having poor prognosis and supported their extraction (Dutner et al., 2020; Gutmann & Rakusin, 1994).
- Presence of signs and symptoms potentially indicating but not confirming pulpal pathoses, such as delayed pulpal response to thermal stimuli (Abou‐Rass, 1983), severe cold sensitivity (Kim et al., 2013), or symptoms persistence after initial management of teeth with RP at baseline (Guthrie & DiFiore, 1991; Kang et al., 2016; Kim et al., 2013; Lee et al., 2021a; 2021b). Contrastingly, other authors did not proceed to endodontic treatment, despite the lack of complete resolution of cold sensitivity after initial management (Ito et al., 1998; Opdam et al., 2008).
- Pulp exposure after removal of cracks or caries (Abbott & Leow, 2009; Kim et al., 2013; Liu & Sidhu, 1995). Nevertheless, it has been demonstrated that direct pulp capping could preserve pulp vitality in case of pulp exposure during caries removal at the crack area (Kanamaru et al., 2017).
- Prosthetic reasons imposing post placement for additional retention of the coronal restoration (Abbott & Leow, 2009).

Besides, it should be acknowledged that a number of studies did not specify why endodontic treatment was required, potentially due to their retrospective nature (Chen et al., 2021; Malentacca et al., 2021). Particularly, with regard to teeth with previous endodontic treatment, the overwhelming majority of researchers provided retreatment (Chen et al., 2021; Davis & Shariff, 2019; de Toubes et al., 2022; Kang et al., 2016; Krell & Caplan, 2018; Nguyen Thi & Jansson, 2021), but did not clarify the exact reasons, for example, whether the teeth had inadequate root canal fillings, developing or persisting periapical pathoses and/or symptoms or if the retreatment was performed for prevention of the above due to secondary caries or the presence of the crack line itself, which could act as a pathway for bacterial invasion. On the other hand, certain investigators proceeded directly to coronal restoration of previously endodontically treated cracked teeth without providing retreatment (Mahgoli et al., 2019; Michaelson, 2015, 2017) while others did not explain whether secondary endodontic treatment was part of their management (Abou‐Rass, 1983; Liao et al., 2022; Malentacca et al., 2021).

### 6.2 Risk factors for pulp survival in cracked teeth with baseline NP/RP

As previously described, restorative protocols during initial management of cracked teeth with NP/RP may influence the likelihood of these requiring endodontic treatment (Wu et al., 2019). However, pulp survival has also been associated with several baseline variables. A brief summary is provided in the following sections.

#### 6.2.1 Crack characteristics

Crack extension and location have been shown as predicting factors of endodontic treatment. Kanamaru et al. (2017) classified the extension of cracks into three categories (middle or deep part of dentine and pulpal involvement) and demonstrated that the deeper the crack the more likely was the need for endodontic treatment. As for crack location, Krell and Rivera (2007) reported that the majority of teeth that needed endodontic treatment (56%) had a crack in the distal marginal ridge. Nevertheless, it was not reported if this effect reached statistical significance. Both ridges were involved in 29% of cases while 15% of teeth presented with mesial cracks.

#### 6.2.2 Probing depth

Baseline presence of deep pocket depth (>6 mm) corresponding to the crack area was another factor linked with a higher risk of endodontic treatment, according to a retrospective study that investigated the outcomes of two different patient cohorts (from 2009 to 2019, respectively) (Lee et al., 2021b). This finding may be explained by the fact that deeper pocket depths have been considered to denote deeper crack extension (Gutmann & Rakusin, 1994). However, it should be mentioned that the findings by Lee et al. (2021b) may be influenced by the inclusion of more severe cases since their sample encompassed both teeth with NP/RP and teeth that required endodontic treatment from baseline (Table 3).

#### 6.2.3 Symptoms

Preoperative pain on percussion significantly increased the possibility of root canal treatment, as demonstrated in the prospective cohort study by Lee et al. (2021a). Pulp survival rates were 46% on teeth with baseline pain on percussion and 94% without this symptom. Although tenderness to percussion usually indicates periapical inflammation, it can also occur in pulpitis due to stimulation of pulpal mechanoreceptors or central sensitization (Owatz et al., 2007).

As for cold hypersensitivity, defined as pain lingering over 10 s after cold stimuli, the above study (Lee et al., 2021a) found no correlation to pulp survival. On the other hand, Lee et al. (2021b) reported a higher risk of endodontic treatment for symptomatic cracked teeth, vaguely defined as those with sensitivity to cold or bite pain, compared to asymptomatic. This discrepancy might stem from the ambiguity of the diagnostic criteria of the latter study regarding cold sensitivity, the inherent subjectivity in the interpretation of sensibility tests as well as the fact that the latter study also included teeth that required endodontic treatment from baseline (Table 3), hence initial symptoms might had been more severe.

#### 6.2.4 Patient gender

Male patients exhibited significantly higher risk of pulpal complications (36%) compared with females (22%) in the retrospective study by Wu et al. (2019). It was hypothesized that the greater risk of males in undergoing endodontic treatment could be attributed to their higher masticatory forces. However, the association was relatively weak while another study found no difference in terms of pulp survival between males and females (Lee et al., 2021a).

### 6.3 Endodontic protocols

The following sections summarize the available data with regard to the protocols implemented during the different stages of root canal treatment in cracked teeth.

#### 6.3.1 Initiation of endodontic treatment

It was generally advised that all previous restorations and caries ought to be removed before endodontic treatment to assess crack location and extent as well as tooth restorability (Abou‐Rass, 1983; de Toubes et al., 2022; Krell & Caplan, 2018; Malentacca et al., 2021). This procedure was facilitated by rubber dam placement, visual magnification, methylene blue staining, or autofluoresence (de Toubes et al., 2020; Gutmann & Rakusin, 1994; Jun et al., 2019; Ritchey et al., 1957). Relative studies also highlighted the necessity for minimum tooth structure removal when managing cracked teeth (de Toubes et al., 2022; Gutmann & Rakusin, 1994; Malentacca et al., 2021). In this respect, some authors chose a conservative (Davis & Shariff, 2019; de Toubes et al., 2022; Fawzy et al., 2020) over a standard access cavity (Sim et al., 2016), since the former was reported to increase fracture strength of endodontically treated teeth due to the preservation of pericervical dentine and part of the pulp chamber roof (Plotino et al., 2017).

#### 6.3.2 Chemomechanical preparation

As for canal enlargement, an earlier investigation reported solely manual instrumentation complemented with Gates–Glidden burs (Gutmann & Rakusin, 1994) whereas more recent studies used nickel‐titanium rotary files (Davis & Shariff, 2019; Lee et al., 2021a; Lu et al., 2021). Other researchers applied both techniques according to case characteristics (Malentacca et al., 2021) or timing of the treatment, as did Krell and Caplan (2018) who changed their instrumentation standards over the 25‐year period of their study. Rotary nickel‐titanium instruments were also employed for the removal of previous obturation materials, when secondary endodontic treatment was performed (Davis & Shariff, 2019).

Irrigation protocol was mainly based on the use of 1%–5.25% sodium hypochlorite solution (Fawzy et al., 2020; Kim et al., 2013; Sim et al., 2016; Tan et al., 2006) with supplementary sonic or ultrasonic activation (Davis & Shariff, 2019; Malentacca et al., 2021). Besides, Lu et al. (2021) combined sodium hypochlorite with 3% hydrogen peroxide, while others used 17% ethylene diamine tetra acetic acid (EDTA) solution for the removal of the smear layer, either as a final (Fawzy et al., 2020) or a penultimate irrigant followed by 2% chlorhexidine (Davis & Shariff, 2019) or 96% ethyl alcohol (Malentacca et al., 2021).

#### 6.3.3 Intracanal medication

There has been no report regarding single‐visit endodontic treatment in relative studies focusing on cracked teeth. In fact, it was described that the root canals were medicated with calcium hydroxide and a second session was arranged after 1‐3 weeks (Davis & Shariff, 2019; de Toubes et al., 2020; Lu et al., 2021; Malentacca et al., 2021). According to Gutmann and Rakusin (1994), application of phenol or formaldehyde‐based medications should be avoided since they could adversely influence the periodontium by diffusing through the crack line. Moreover, in a case where symptoms persisted a week after calcium hydroxide placement, simvastatin was used empirically as an intracanal medicament for a 3‐month period due to its antibacterial, antioxidant, anti‐inflammatory as well as bone healing properties (Fawzy et al., 2020). The results showed complete resolution of signs and symptoms after 1 week, while the tooth remained functional with normal clinical and radiographic appearance at the 12‐month recall.

#### 6.3.4 Obturation

Root canals were obturated with gutta‐percha along with an epoxy resin (Fawzy et al., 2020; Lu et al., 2021) or a zinc oxide‐eugenol sealer (Davis & Shariff, 2019; Krell & Caplan, 2018; Sim et al., 2016; Tan et al., 2006). Although some investigators performed lateral condensation (Fawzy et al., 2020; Krell & Caplan, 2018), the majority chose a thermoplastisised gutta‐percha technique (de Toubes et al., 2020; Kim et al., 2013; Lee et al., 2021a; Lu et al., 2021; Malentacca et al., 2021) as it was maintained that, especially in the canal associated with the crack, lateral forces should be eliminated to prevent further crack propagation (Gutmann & Rakusin, 1994). Likewise, it was also supported that, regardless of the obturation method, excessive condensation (Abou‐Rass, 1983; Lu et al., 2021) as well as engagement of the pluggers into the root canal walls (Gutmann & Rakusin, 1994; Malentacca et al., 2021) should be avoided.

#### 6.3.5 Post‐operative instructions

Strict adherence to specific post‐operative instructions has been considered as a crucial part of the management of cracked teeth requiring endodontic treatment. Relative protocols reported in the literature included the use of analgesics to manage postoperative pain, optimal oral hygiene, communication with the operating dentist in case of any discomfort (Lu et al., 2021), soft diet (Davis & Shariff, 2019; Lu et al., 2021), and avoidance of chewing on the site of the affected tooth until the placement of permanent postendodontic restoration (Davis & Shariff, 2019; Gutmann & Rakusin, 1994).

### 6.4 Concluding remarks

The following conclusions can be drawn regarding endodontic treatment in cracked teeth.

- A lack of consensus can be observed regarding the indications for endodontic treatment. It is therefore essential that standardized criteria be developed to guide the decision‐making process.
- The risk factors for pulp survival need to be validated via randomized trials, since relevant data derive from a limited number of observational studies.
- The majority of cracked tooth studies did not provide documentation as for their endodontic protocols. Future research should assess the impact of intra‐operative endodontic variables and especially recent endodontic advancements on the outcomes of cracked teeth.

## 7 RESTORATIVE APPROACHES FOR CRACKED TEETH REQUIRING ENDODONTIC TREATMENT

Current restorative approaches for cracked teeth requiring endodontic treatment can be divided into the interim (intra‐operative or post‐endodontic) and the definitive post‐endodontic restorations (Table 2).

### 7.1 Interim coronal restorations

#### 7.1.1 Intra‐operative

Intra‐operative interim restorations were advised to stabilize tooth segments and prevent further crack propagation during endodontic procedures (Liu & Sidhu, 1995; Ritchey et al., 1957). The most commonly performed intra‐operative interim treatment comprised extra‐coronal splinting, which was provided either in the form of temporary crowns (Lee et al., 2021b; Liao et al., 2022; Ritchey et al., 1957) or orthodontic bands (Gutmann & Rakusin, 1994; Liao et al., 2022; Liu & Sidhu, 1995). Besides, Malentacca et al. (2021) proceeded to pre‐endodontic reconstruction in case of heavily compromised cracked teeth. Between endodontic sessions, other authors preferred direct intra‐coronal restorations using composite resin (de Toubes et al., 2020, 2022) or temporary filling materials (de Toubes et al., 2022; Fawzy et al., 2020; Lu et al., 2021). In addition to the aforementioned methods, occlusal adjustment was suggested to protect the cracked tooth from excessive masticatory forces (de Toubes et al., 2020, 2022; Fawzy et al., 2020; Gutmann & Rakusin, 1994; Mahgoli et al., 2019; Malentacca et al., 2021) while a stabilization splint was provided in the presence of parafunctional habits (Liu & Sidhu, 1995).

#### 7.1.2 Post‐endodontic

Apart from immobilizing the cracked tooth and averting additional crack progression, post‐endodontic interim treatment was employed to provide adequate time for complete resolution of symptoms before embarking on the final restoration (Kim et al., 2013; Lee et al., 2021a). For that purpose, both provisional crowns (Kang et al., 2016; Kim et al., 2013; Lee et al., 2021a) and orthodontic bands (Kang et al., 2016) were employed, although relevant studies did not mention the exact period that those remained so that the next stage of approach could be decided. Instead, they vaguely reported that permanent treatment could be performed once cracked teeth became asymptomatic.

By contrast, it has been maintained that prolonged interim treatment may endanger the prognosis of cracked teeth, as provisional restorations rarely provide sufficient protection from occlusal forces (Guthrie & DiFiore, 1991) and furthermore, they might dislodge resulting in microleakage and deeper crack extension (Wu et al., 2019). That was apparently the reason why other groups of researchers (Davis & Shariff, 2019; de Toubes et al., 2020; de Toubes et al., 2022; Lu et al., 2021; Malentacca et al., 2021) proceeded directly to definitive cuspal coverage restorations right after completing the endodontic intervention. Nevertheless, even when a post‐endodontic interim restoration was not performed, the time interval between endodontic treatment and permanent restoration exhibited a considerable range. Some studies reported placement of chairside CAD/CAM crowns or onlays at the same appointment (de Toubes et al., 2020; de Toubes et al., 2022), whereas others reported that definitive restorations were delayed for more than 6 weeks, especially when different operators had to cooperate for the endodontic‐restorative management (Davis & Shariff, 2019).

### 7.2 Definitive post‐endodontic restorations

**Figure 4:**
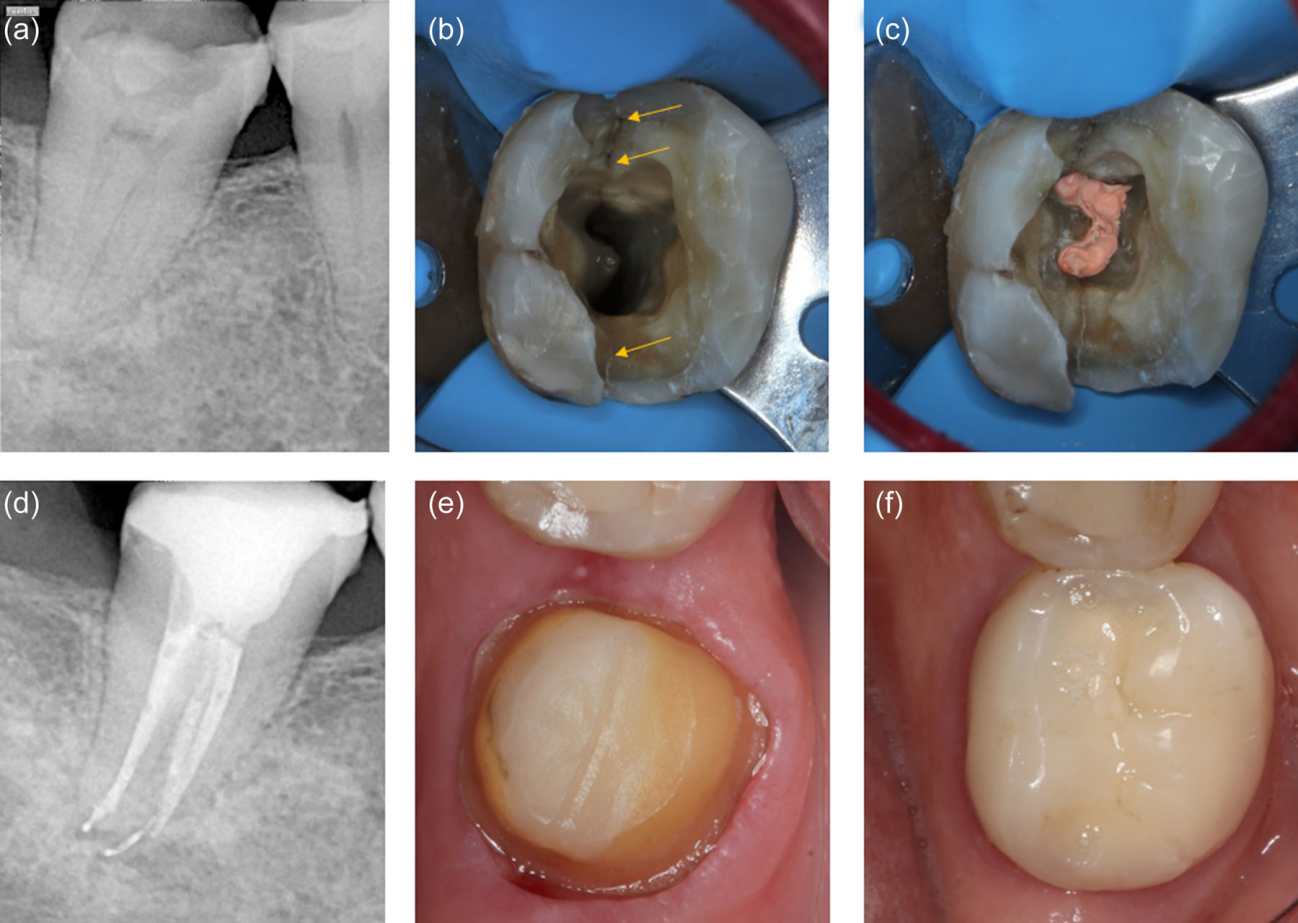
Treatment sequence of a cracked 46 with irreversible pulpitis (a) preoperative long‐cone periapical radiograph (b) access cavity for endodontic treatment revealing mesial and distal crack lines (arrows) and C‐shaped canal configuration (c) root canal obturation completed (d) periapical radiograph after completion of endodontic treatment (e) crown preparation (f) occlusal view after cementation of a metal ceramic crown.

**Figure 5:**
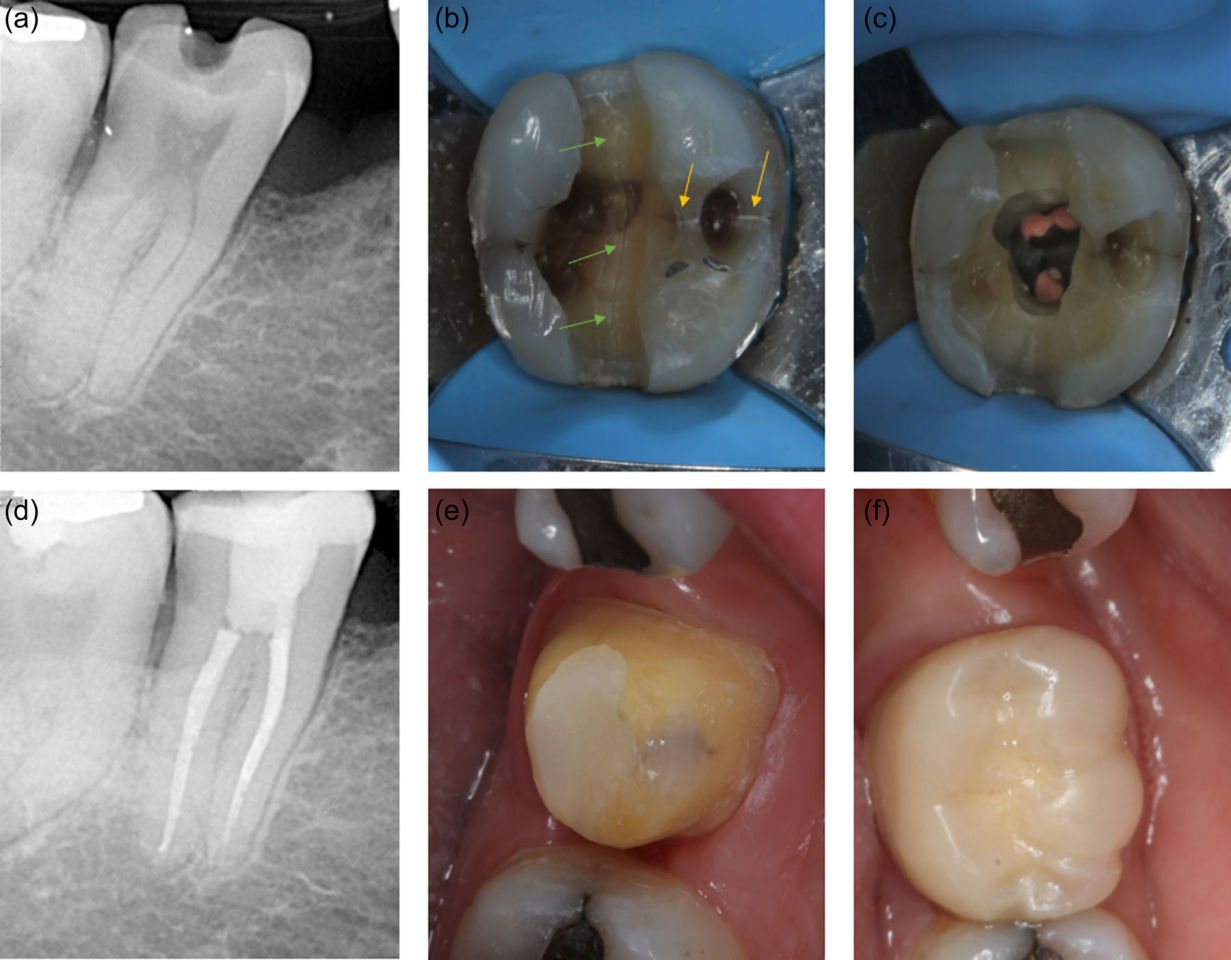
Treatment sequence of a cracked 47 with irreversible pulpitis (a) preoperative long‐cone periapical radiograph (b) removal of previous amalgam restoration revealing multiple crack lines running towards mesiodistal (green arrows) and buccolingual direction (yellow arrows) (c) root canal obturation completed (d) periapical radiograph after completion of endodontic treatment (e) crown preparation (f) occlusal view after cementation of a metal ceramic crown.

The vast majority of clinical studies reported crowns as permanent post‐endodontic restorations for cracked teeth (Table 2) (Figures 4 and 5) with a broad variation regarding materials, including metal‐ceramic (Liu & Sidhu, 1995; Mahgoli et al., 2019), full ceramic (de Toubes et al., 2020, 2022; Lee et al., 2021a; Lu et al., 2021) and full metal (Ito et al., 1998; Jun et al., 2019; Kanamaru et al., 2017; Lee et al., 2021a; Liu & Sidhu, 1995), although most investigators did not share specific details as for the crown material (Davis & Shariff, 2019; Kim et al., 2013; Lee et al., 2021b; Sim et al., 2016; Tan et al., 2006). What is more, given the etiological relationship between cracks and occlusal interferences (de Toubes et al., 2022; Kanamaru et al., 2017), it was suggested that definitive full coverage restorations receive meticulous occlusal adjustment (Davis & Shariff, 2019; Gutmann & Rakusin, 1994) with minimal centric and no excursive contacts (Abou‐Rass, 1983). Despite these indications, Davis and Shariff (2019) described that 78.7% of the definitive crowns were found to have premature occlusal contacts at the review appointment.

**Figure 6:**
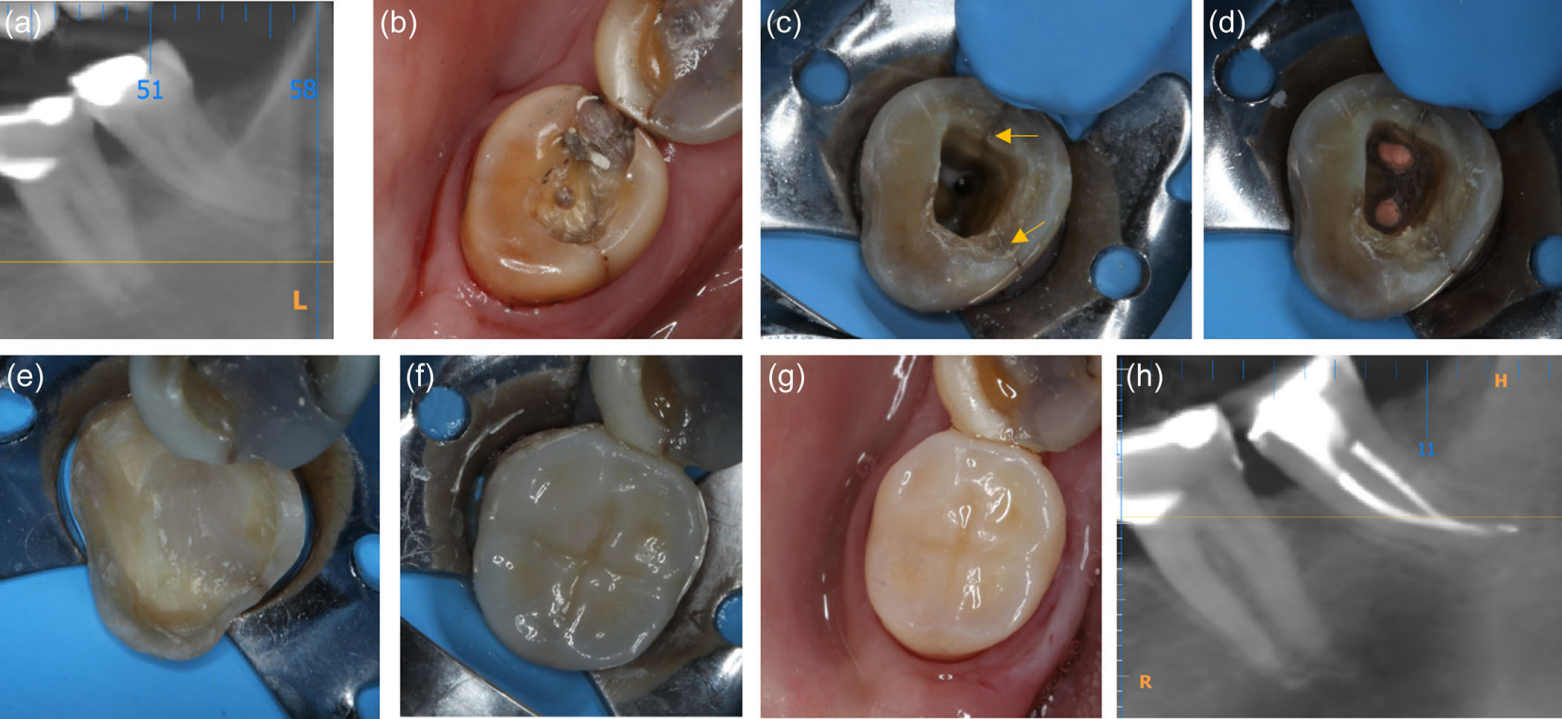
Treatment sequence of a cracked 38 with irreversible pulpitis (a) preoperative cone beam computed tomography (CBCT) (b) occlusal view after removal of previous amalgam restoration (c) cuspal reduction and access cavity for endodontic treatment with visible cracks at mesial and lingual walls (arrows) (d) root canal obturation completed (e) preparation for indirect onlay restoration (f) indirect composite onlay bonded (g and h) postoperative occlusal view and CBCT image.

Alternatives to crowns included direct composite restorations (Lu et al., 2021; Malentacca et al., 2021; Nguyen Thi & Jansson, 2021) and indirect onlays (de Toubes et al., 2022) (Figure 6). Regarding endocrowns, there is a lack of clinical evidence since relevant data derive from laboratory studies (Anton Y Otero et al., 2021; Lin et al., 2013) (Table 4).

Regardless of the definitive treatment being a crown (Davis & Shariff, 2019; Gutmann & Rakusin, 1994; Liu & Sidhu, 1995) or a direct cuspal coverage composite restoration (Malentacca et al., 2021), intraorifice barriers have been frequently implemented as part of the core build‐up. The rationale for this approach is that it can obtain a superior seal of the crack line compared to that provided by gutta‐percha (Pisano et al., 1998) and increase the radicular fracture resistance (Nagas et al., 2010). Various materials have been employed for this technique; earlier reports reported using amalgam (Liu & Sidhu, 1995) or a mixture of amalgam and glass‐ionomer cement (Gutmann & Rakusin, 1994) while more recent studies reported microscope‐assisted placement of resin composite (Davis & Shariff, 2019; Malentacca et al., 2021). Another method that has been shown to enhance the fracture strength of endodontically treated cracked teeth restored with crowns is the annular placement of polyethylene fibers as a core reinforcement, although current evidence solely relies on laboratory data (Shi et al., 2021) (Table 4).

### 7.3 Concluding remarks

The following points can be highlighted regarding the restorative approaches for cracked teeth requiring endodontic treatment:

- There is no conclusive evidence whether treatment outcomes could be enhanced by an interim treatment before proceeding to the definitive post‐endodontic restoration. Moreover, the ideal type as well as the duration of the interim treatment ought to be established.
- A general trend can be observed in the literature towards providing crowns as definitive post‐endodontic restorations, whereas the evidence regarding less invasive types of coronal restorations (direct restorations, indirect onlays, endocrowns), which could presumably be more beneficial for the structural integrity of cracked teeth, is scarce.
- Intra‐orifice barriers and annular fiber reinforcement have been suggested as part of the core build‐up to improve the fracture resistance of endodontically treated cracked teeth.

## 8 OUTCOMES OF ENDODONTICALLY TREATED CRACKED TEETH

A large variance can be noted in the evaluation of outcomes of endodontically treated cracked teeth (Table 2), as some studies reported only tooth survival (Kang et al., 2016; Liao et al., 2022; Nguyen Thi & Jansson, 2021; Sim et al., 2016; Tan et al., 2006), others focused on treatment success (Chen et al., 2021; Krell & Caplan, 2018) while a number of researchers examined both variables (Davis & Shariff, 2019; de Toubes et al., 2022; Malentacca et al., 2021).

### 8.1 Survival rates

By and large, tooth survival after endodontic treatment was recorded if a cracked tooth was present in the dental arch at the time of review (de Toubes et al., 2022; Kang et al., 2016; Malentacca et al., 2021; Nguyen Thi & Jansson, 2021; Sim et al., 2016; Tan et al., 2006), whereas other authors (Davis & Shariff, 2019; Liao et al., 2022) applied more stringent standards since they considered a tooth as “survived,” when it was also functional and asymptomatic, although the latter criteria are more indicative of treatment success, as it will be described later. “Survival” has been advocated as preferable to “success” when assessing the outcomes of cracked teeth after endodontic treatment, because it enables direct comparison to alternative treatment modalities, such as dental implants, since relative studies mostly investigated survival rather than success (Leong et al., 2020; Sim et al., 2016).

So far there have been two systematic reviews with meta‐analyses exploring cracked teeth survival following endodontic treatment (Leong et al., 2020; Olivieri et al., 2020). Overall, both reviews described survival rates between 84.1% and 88% at 12–60 months after intervention, which are comparable to those illustrated for endodontically treated teeth in general (86%–93% over 2–10 years) (Ng et al., 2010), implying that root canal treatment can be a viable treatment method when a crack is present.

Regarding data released after the publication of the above systematic reviews, de Toubes et al. (2022) described that 57 of 63 endodontically treated cracked teeth (90.5%) survived after a mean 3.3‐year observation period whereas, Nguyen Thi and Jansson (2021) reported decreased survival rates (68% and 54% after 5 and 10 years, respectively), probably because treatment was performed by general dentists, who apparently lacked high expertise or worked without magnification. Between studies which focused on cracks with radicular extension, Malentacca et al. (2021) found reduced survival (68% in a mean 67‐month observation period) compared to Davis and Shariff (2019) (96.6% after 4 years), even though the latter study adopted more stringent definition criteria, as mentioned above. Although both research teams employed intra‐orifice resin barriers in the post‐endodontic restoration, Davis and Shariff (2019) proceeded to an expeditious crown provision while Malentacca et al. (2021) performed direct cuspal coverage composite restorations. In addition, Davis and Shariff (2019) employed a stricter post‐endodontic protocol with specific postoperative instructions and regular reviews.

Besides, a common downward trend in cracked teeth survival has been pointed out at extended observation periods (Davis & Shariff, 2019; de Toubes et al., 2022; Leong et al., 2020; Liao et al., 2022; Malentacca et al., 2021), which admittedly corresponds to cracks propagating deeper over time, leading eventually to tooth loss.

### 8.2 Success rates

When it comes to a case deemed “successful,” there is an even wider heterogeneity recorded in the literature. According to the consensus report of the European Society of Endodontology (2006), root canal treatment should be assessed at least after 1 year, with findings representing a favorable (successful) outcome being: absence of pain, swelling, and other symptoms, no sinus tract, no loss of function and radiographic evidence of a normal periodontal ligament space around the root. Lesions that have remained unchanged or only diminished in size are considered to have an ‘uncertain’ outcome and warrant further observation over up to 4 years.

Despite the fact that some investigators conformed to the aforementioned guidelines (Krell & Caplan, 2018; Malentacca et al., 2021) others evaluated success only by disappearance of symptoms (Kim et al., 2013; Lee et al., 2021b) and reduction in the size of the periapical lesion (Liu et al., 2021). On the contrary, a broader range of prerequisites for success was implemented by other research teams, which further considered no increase in crack‐associated periodontal probing (Davis & Shariff, 2019; de Toubes et al., 2022), periapical index (PAI) score ≤2 (de Toubes et al., 2022; Dow, 2016), no increase in crestal bone loss, no sensitivity in percussion, palpation or bite test, as well as the presence of an occlusally, equilibrated full‐coverage restoration (Davis & Shariff, 2019). Respectively, two studies recorded as “failures” cases with an unchanged periapical lesion, although they were followed up for a mean period of 19 months (Liu et al., 2021) and 23.3 months (Chen et al., 2021), instead of the proposed 4‐year time span (Endodontology, 2006). In addition, other studies (Lee et al., 2021b; Lu et al., 2021) had a shorter follow‐up than the minimum required (1 year) for the assessment of success (Endodontology, 2006).

Between the two available systematic reviews, only Olivieri et al. (2020) provided information for success and demonstrated that 82% of endodontically treated cracked posterior teeth were deemed “successful” at 1‐year recall. Nevertheless, their results should be interpreted with extreme caution as there was no specific explanation for their definition of “success.”

As regards to studies published after the above systematic review, Chen et al. (2021) reported a decline in success rates (75.8% after a mean 23.3‐month follow‐up) which was potentially because of the fact that treatment was provided in a postgraduate clinic, 15/62 of the samples lacked a permanent coronal restoration and as earlier mentioned, more limiting criteria for success were imposed. Similarly, reduced success rates were found by Malentacca et al. (2021) (53% after 5 years) possibly due to focusing on teeth with radicular cracks. On the other hand, the superior outcomes reported by Davis and Shariff (2019) (90.6% 2‐ to 4‐year success along with an average reduction of 0.41 mm in probing depths at the site of the crack), despite also assessing cracks with radicular extension as well as having established more stringent criteria for success, might be attributed to their specific post‐endodontic treatment protocol, as described in the above section regarding survival. In addition, the latter study is advantageous in terms of being prospective with a relatively high recall rate (81.5%).

### 8.3 Prognostic factors for the outcomes of endodontically treated cracked teeth

In the following sections, it was attempted to group the most predominant predicting variables for the outcomes of endodontically treated cracked teeth. Nonetheless, it should be acknowledged that the substantial diversity of related studies as for the strategy employed to measure treatment outcomes (survival and/or success), could potentially influence the prevalence of certain prognostic factors. For example, a factor that marginally lost significance in a statistical model exploring survival, might hypothetically have proven to be significant, if more constricting criteria for success were applied and thus, more failures were estimated.

#### 8.3.1 Crack extension

The extent of the crack is an important factor when considering the prognosis of a root‐filled cracked tooth (Malentacca et al., 2021; Sim et al., 2016). The exact mechanism is likely to involve the fact that deep cracks are rarely encompassed by the restoration margins and consequently, they become recontaminated or gradually propagate to complete fractures (Leong et al., 2020). What is more, the attachment apparatus alongside the crack line may breakdown triggering periodontal implications (Malentacca et al., 2021).

It should be highlighted that the variable of the crack extent might have been underestimated, as a number of investigations did not provide treatment for teeth having cracks extending to the pulp chamber floor (Gutmann & Rakusin, 1994; Krell & Rivera, 2007; Liu & Sidhu, 1995; Ritchey et al., 1957) or beyond the root canal orifices (Chen et al., 2021), especially when a periodontal pocket ≥4 mm was co‐existent (de Toubes et al., 2022). Besides, crack extension was estimated with different criteria among relative studies. For example, cracks have been categorized as:

- Radicular if extended to the pulpal floor or beyond the orifices and coronal if confined within the pulp chamber walls (Sim et al., 2016; Tan et al., 2006).
- Proximal radicular, when extending up to the coronal third of root, and deep radicular, when extending to the middle or apical root thirds (Malentacca et al., 2021).
- Supragingival or subgingival (Kang et al., 2016; Liao et al., 2022).

Specifically, in a study by Sim et al. (2016), multivariable analysis found that radicular cracks, increased the odds of tooth loss by 11‐fold when compared to coronal cracks, with other variables being held constant. However, a previous study by the same research team (Tan et al., 2006) did not confirm crack extension to be of significance regarding tooth prognosis, probably due to its smaller sample size (50 vs. 84 teeth) and shorter follow‐up period (2 vs. 5 years), implying that more failures would be recorded in a longer observation period as longitudinal fractures are expected to progress deeper over time. Accordingly, Kang, who also had a 2‐year follow‐up period but assessed crack extent distinctively, did not demonstrate a significant difference between supragingival or subgingival cracks. A recent systematic review (Leong et al., 2020), which took into account the aforementioned three studies (Kang et al., 2016; Sim et al., 2016; Tan et al., 2006), estimated 8.9% lower risk of extraction for coronal cracks compared to radicular, but the difference had only possible clinical value and not statistical significance. This could be attributed to the considerable heterogeneity of their included studies in terms of categorizing crack extent, as subgingival cracks were grouped with radicular, although these do not necessarily coincide with each other.

Moreover, considering the limitations of an in vitro study (Lin et al., 2013), it was remarked that the failure risk for fractures extending below bone level and to the mid‐root area was higher than cracks extending up to 1 mm above bone level, regardless of the type of restoration (ceramic onlay, endocrown, and crown) and under the same load conditions.

When it comes to studies that included only radicular fractures, Malentacca et al. (2021) proved that proximal radicular cracks were associated with significantly higher 5‐year survival rates compared with deep radicular cracks (78% and 58%, respectively), according to the definitions previously described. Furthermore, teeth with deep radicular cracks were more frequently associated with a probing defect and lower bone recovery after therapy. Nevertheless, another relevant study (Davis & Shariff, 2019) demonstrated no difference for different depths of radicular cracks in relation to treatment success. Apart from differences in the treatment protocol, this could be also due to the possibility that some deep radicular cracks were miscalculated since it is difficult to track their extent along the root, especially in curved canals, even with the use of a dental microscope.

#### 8.3.2 Pre‐treatment periodontal pocket

A pre‐treatment periodontal pocket is another commonly demonstrated factor to significantly affect tooth survival (Leong et al., 2020; Malentacca et al., 2021; Olivieri et al., 2020) as well as success (Krell & Caplan, 2018) of orthograde endodontic treatment in cracked teeth. The systematic review by Olivieri et al. (2020) revealed that, in the presence of a periodontal pocket, the risk of extraction surged by 11% and when this variable was absent, the 1‐year survival rate of endodontically treated cracked teeth increased to 97%. Since patients did not have any periodontal disease as an inclusion criterion in this review, it could be supported that the periodontal defect was caused by the crack extension into the root surface itself, acting as a pathway for bacterial invasion (Abou‐Rass, 1983; Gutmann & Rakusin, 1994).

What it is worth emphasizing is the heterogeneity in the cut‐off points applied by different studies when evaluating periodontal probing. Some authors investigated pockets ≥4 (de Toubes et al., 2022; Kanamaru et al., 2017), others ≥5 mm (Chen et al., 2021; Davis & Shariff, 2019; Krell & Caplan, 2018; Liao et al., 2022), or >6 mm (Kang et al., 2016). That was probably the reason why the systematic review by Leong et al. (2020) failed to find statistical significance but showed only clinical association between pretreatment periodontal probing and risk of extraction, as it included only two papers (Sim et al., 2016; Tan et al., 2006) for final analysis, which both examined pockets >3 mm.

Additional factors for certain studies indicating deep periodontal probing to be statistically insignificant but with potential clinical value as for treatment success, could be the exclusion of teeth with radicular cracks (cracks extending to pulp chamber floor or beyond the orifices) (Chen et al., 2021), which are more likely to induce periodontal defects (Malentacca et al., 2021), or the inclusion of a restrictive range of pocket depths (up to 7 mm) (Davis & Shariff, 2019), which potentially excluded more catastrophic fractures.

#### 8.3.3 Definitive post‐endodontic restoration

Endodontically treated cracked teeth with no permanent restoration to replace the temporary filling are proved to have an increased risk of failure by 12.5‐fold compared to those receiving crowns (Chen et al., 2021). This reported risk is superior to the relevant findings for endodontically teeth in general (4–6‐fold risk) (Ng et al., 2010), presumably due to the stricter criteria applied for successful cases as well as the presence of the crack line itself, which additionally weakens the remaining tooth structure.

When comparing crowns to direct composite as post‐endodontic restorations in cracked teeth with irreversible pulpitis, the former group was related to having a better therapeutic effect (efficacy was evaluated by the absence of pain, gingival swelling, surrounding tissue inflammation, chewing discomfort, and periapical radiolucency), increased bite force and chewing efficiency, improved quality of life, as well as reduced periodontal index (composed of the plaque index, probing depth, gingival sulcus bleeding index and gingival index) in a 6‐month follow‐up and the differences, were statistically significant (Lu et al., 2021). Accordingly, Nguyen Thi and Jansson (2021) reported a significantly higher risk of extraction when cracked teeth received composite restorations in comparison to full crowns following endodontic therapy. However, an important factor to consider when evaluating the relatively high survival rates of teeth treated with crowns (95% after 10 years) as opposed to the overall ones (54%) in the latter study, is that treatment was performed by general dentists who probably felt more confident to invest in a full crown when treating easier cases with conceivably more predictable outcomes.

With regard to comparisons between crowns and onlays, de Toubes et al. (2022) noted that onlays had significantly higher correlation to tooth loss, although their sample included both cracked teeth that required endodontic treatment as well as teeth that remained vital. Besides, laboratory studies that used endodontically treated maxillary cracked premolar models presented heterogeneous results (Table 4). Lin et al. (2013) highlighted that after providing metal post and composite build‐up, onlays exhibited higher failure probabilities for different crack depths compared to both crowns and endocrowns. On the other hand, Shi et al. (2021) showed no statistically significant differences between crowns and onlays, although crowns resulted in superior fracture resistance when both combined with an annular fiber‐reinforced base. As for fracture pattern though, fiber‐reinforced onlays exhibited significantly more favorable failures (fractures above or about 1 mm below cementoenamel junction) compared with crowns.

As regards to endocrowns, Lin et al. (2013) remarked that they provided comparable fracture resistance to crowns in cases of shallow cracks (about 1 mm above bone level), but they showed a higher failure risk for deep cracks (below bone level to mid‐root), especially under increased occlusal forces (above 250 N). Furthermore, a fiber‐reinforced base did not significantly affect the fracture resistance of teeth restored with endocrowns, but it resulted in more fractures being restorable (50%–80%) in comparison to the control group (30%), which included a base of flowable composite (Anton Y. Otero et al., 2021). It should be mentioned though that both the above in vitro studies did not evaluate the effect of lateral forces, which are crucial for cracked teeth from a clinical point of view (de Toubes et al., 2022; Kanamaru et al., 2017).

Restoring cracked teeth by post placement was also underlined to significantly decrease survival rates (Chen et al., 2021; de Toubes et al., 2022), possibly because samples receiving posts are usually more compromised than their counterparts and the tooth preparation for the postplacement may further weaken tooth structure. This finding was in agreement with previous treatment recommendations by other authors (Abou‐Rass, 1983; Gutmann & Rakusin, 1994; Liu & Sidhu, 1995). However, it was confirmed that the placement of a definitive full crown acts protectively by reducing postrelated risk of tooth loss (de Toubes et al., 2022).

#### 8.3.4 Pulpal diagnosis

Berman and Kuttler (2010) demonstrated that in the absence of caries, restorations, or luxation injuries, pulp necrosis is likely to be caused by a crack extending from the occlusal surface into the pulp and they described this condition as ‘fracture necrosis’. They recommended that in such cases extraction should be considered as the primary treatment option, since retention of cracked teeth with non‐vital pulps would potentially induce extensive periodontal and/or apical bone loss complicating future placement of an implant or a fixed bridge. Although that concept was in accordance with the treatment strategy adopted by other authors (Dutner et al., 2020; Gutmann & Rakusin, 1994), only a single investigation managed to demonstrate that the loss of pulp vitality impaired the 2‐year prognosis of cracked teeth (Liao et al., 2022). Contrastingly, the vast majority of literature reports no significant correlation between pulpal diagnosis and risk of extraction (Kang et al., 2016; Leong et al., 2020; Malentacca et al., 2021; Nguyen Thi & Jansson, 2021; Olivieri et al., 2020; Sim et al., 2016) or treatment failure in cracked teeth (Davis & Shariff, 2019; Krell & Caplan, 2018).

#### 8.3.5 Radiographic findings

From a radiographic perspective, samples with a pre‐treatment periradicular radiolucency were found to have a 27.5% lower success rate than the negative group (*p* = .01) within a mean 23.3‐month follow‐up period (Chen et al., 2021). That was in agreement with the findings from another study, which compared data from 2009 to 2019 and confirmed that, in both time points, cracked teeth with apical lesions had significantly higher rates of persistent symptoms after 3 and 6 months (Lee et al., 2021b).

Respectively, when a periapical diagnosis of chronic apical periodontitis, suppurative apical periodontitis, or acute apical abscess was made, 1‐year success rates of orthograde endodontic treatment in cracked teeth plunged by 11% as compared with the group diagnosed with normal periapical tissues or acute apical periodontitis (Krell & Caplan, 2018). In fact, the latter authors employed three factors deemed as the most predictive of success (periradicular diagnosis, distal marginal crack, and periodontal probing ≥5 mm) to generate a prognostic index known as the “Iowa Index,” to guide practitioners' decision making regarding a cracked tooth that requires endodontic treatment. Nevertheless, the fact that a previous diagnostic terminology was applied, degrades the potential clinical value of the aforementioned index. Furthermore, despite the large sample size (*n* = 1406) and the long enrollment period (25 years) recorded in that study, having a small recall rate (27%), as well as a short follow‐up period (1 year), were considered as additional weaknesses.

By contrast, other studies showed that neither the pretreatment periradicular diagnosis (Davis & Shariff, 2019; Sim et al., 2016) nor the existence of a preoperative periapical lesion (Davis & Shariff, 2019; Malentacca et al., 2021) was of statistical significance towards treatment outcomes. Besides, Malentacca et al. (2021) found further bone loss, as evaluated radiographically 1 year after treatment, to be a significant risk factor for extraction, although it was unclear whether they assessed periapical and/or periodontal bone loss.

#### 8.3.6 Previous endodontic treatment

The majority of existing evidence supports that prior root canal treatment does not influence the outcomes of cracked teeth. Particularly, Krell and Caplan (2018) reported decreased success of previously endodontically treated teeth (74%) compared to other pulpal diagnoses (85% for teeth with irreversible pulpitis and 80% for teeth with pulpal necrosis), however, the differences did not reach statistical significance. No correlation between endodontic treatment before crack development and risk of cracked tooth loss was also confirmed by Malentacca et al. (2021), although they took into account different types of previous endodontic management (endodontic treatment, retreatment and surgical treatment). Given that this was the only study to assess the variable of preceding surgical endodontic treatment in cracked teeth, it should be underlined that all of the three relevant cases failed within 3 years. In addition to the above studies, three further investigations (Chen et al., 2021; de Toubes et al., 2022; Liao et al., 2022) evaluated the variable of prior endodontic intervention, but the reliability of their findings may have been compromised by their limited sample (up to five previously endodontically treated cracked teeth). Among them, solely de Toubes et al. (2022) showed that former root canal treatment was a risk factor for extraction.

Regarding the treatment strategy for previously endodontically treated cracked teeth, the presence of tooth fractures and cracks has been reported not to significantly affect the outcomes of endodontic retreatment (Ng et al., 2011b), implying that this can be an effective treatment approach when indicated.

#### 8.3.7 Terminal abutments

Terminal abutments, defined as the most posterior teeth in the dental arch, were associated with 96% higher risk of tooth loss than those located more anteriorly according to a prospective study investigating the outcomes of nonsurgical root canal treatment in both cracked and noncracked teeth (Ng et al., 2011a).

As for studies focusing solely on cracked teeth, Tan et al. (2006) also found that terminal tooth location was a significant prognostic factor regarding 2‐year survival after endodontic treatment. However, a subsequent study by the same research team, which employed a larger sample (84 teeth) and a longer observation period (5 years), did not manage to find any correlation (Sim et al., 2016). Moreover, the systematic review and meta‐analysis by Leong et al. (2020) described an 8% greater risk of extraction for terminal cracked teeth, although the difference was not statistically significant. Similarly, most of the studies that investigated the variable of terminal tooth position, confirmed no existing association with tooth loss (Davis & Shariff, 2019; Kang et al., 2016; Liao et al., 2022; Olivieri et al., 2020).

#### 8.3.8 Other factors

Additional prognostic factors, such as preoperative presence of multiple cracks (Tan et al., 2006), crack involving the distal marginal ridge (Krell & Caplan, 2018), Class II cavities (Kang et al., 2016), grade I and II mobility as well as spontaneous or palpation pain (Liao et al., 2022), were significantly correlated to cracked tooth outcomes, although those results were provided by isolated studies and apparently, should be validated by future relative investigations.

### 8.4 Concluding remarks

It may be broadly deduced that endodontic treatment can lead to encouraging clinical outcomes of cracked teeth, although several prognostic factors can affect treatment results. However, the limitations of the available evidence should be highlighted; most studies are observational, lack long‐term results and diverge significantly in multiple levels (definition of outcomes, study designs, inclusion criteria, post‐endodontic restorative protocols). Moreover, a gap in the existing literature needs to be emphasized regarding the exploration of intra‐operative prognostic variables, such as apical patency, irrigation protocol, root filling extension, inter‐appointment flare‐up, and iatrogenic complications.

## 9 GENERAL CONCLUSION

It is evident that the literature regarding cracked teeth involves conflicting points while most researchers established their treatment strategy on personal preference rather than universally agreed protocols. Within the limitations of the available studies, it can be inferred that cracked teeth diagnosed with NP/RP can exhibit high pulp and tooth survival rates by the provision of single‐stage treatment with direct or indirect composite restorations while multiple‐stage treatment may be advantageous when a crown definitive restoration is planned. Besides, restoration may not always be indicated, as recent data favor monitoring for certain types of cases, especially in the absence of symptoms or compromised tooth structure. When cracked teeth require endodontic intervention, current evidence suggests that endodontic treatment along with appropriate restorative management may produce outcomes that are comparable to those of non‐cracked root‐filled teeth. However, considering the presence of various predicting factors with respect to the clinical outcomes, treatment planning should be established on a case‐by‐case basis and according to a patient‐centered decision‐making process.

Future research should therefore be directed towards randomized controlled trials to illuminate aspects that remain ambiguous and guide the decision making as for the management of cracked teeth.

## AUTHOR CONTRIBUTIONS

**Angeliki Kakka**: Conceptualization, methodology, investigation, resources, data curation, writing—original draft, writing—review, and editing. **Dimitrios Gavriil**: Conceptualization, methodology, investigation, resources, data curation, writing—original draft, writing—review, and editing. **John Whitworth**: Supervision, writing—review, and editing.

## CONFLICTS OF INTEREST

The authors declare no conflicts of interest.

## Supporting information

Supporting information.

Supporting information.

## Data Availability

Data sharing is not applicable to this article as no datasets were generated or analyzed during the current study.
