# Supplementary material for: Treatment of cracked teeth: A comprehensive narrative review
Source: Clin Exp Dent Res. 2022 Jul 9;8(5):1218–48. doi: 10.1002/cre2.617 (PMC9562569; doi:10.1002/cre2.617)
Supplement: Supplementary file 1 — Supporting information. [file CRE2-8-1218-s002.docx]

| **SUPPORTING INFORMATION 1.**  Distribution of the 64 included studies according to study type | |
| --- | --- |
| **Systematic reviews (n=2)** | |
| Leong *et al.* (2020) | Olivieri *et al.* (2020) |
| **Interventional studies (n=4)** | |
| Davis and Overton (2000) | Opdam and Roeters (2003) |
| Lu *et al.* (2021) | Opdam *et al.* (2008) |
| **Observational studies (n=35)** | |
| Abbott (2001) | Kim *et al.* (2013) |
| Abbott and Leow (2009) | Krell and Caplan (2018) |
| Abou-Rass (1983) | Krell and Rivera (2007) |
| Banerji *et al.* (2014) | Lee *et al.* (2021a) |
| Brynjulfsen *et al.* (2002) | Lee *et al.* (2021b) |
| Cameron (1976) | Liao *et al.* (2022) |
| Chana *et al.* (2000) | Liu *et al.* (2021) |
| Chen *et al.* (2021) | Malentacca *et al.* (2021) |
| Davis and Shariff (2019) | Marchan *et al.* (2013) |
| de Toubes *et al.* (2022) | Ng *et al.* (2011a) |
| Dow (2016) | Ng *et al.* (2011b) |
| Ferracane *et al.* (2022) | Nguyen Thi and Jansson (2021) |
| Guthrie and DiFiore (1991) | Roh and Lee (2006) |
| Hilton *et al.* (2020a) | Signore *et al.* (2007) |
| Hilton *et al.* (2020b) | Sim *et al.* (2016) |
| Homewood (1998) | Tan *et al.* (2006) |
| Kanamaru *et al.* (2017) | Wu *et al.* (2019) |
| Kang *et al.* (2016) |  |
| **Case reports/series (n=17)** | |
| Batalha-Silva *et al.* (2014) | Jun *et al.* (2019) |
| Bearn *et al.* (1994) | Liebenberg (1996) |
| de Toubes *et al.* (2020) | Liu and Sidhu (1995) |
| Dutner *et al.* (2020) | Mahgoli *et al.* (2019) |
| Ehrmann and Tyas (1990) | Michaelson (2015) |
| Fawzy *et al.* (2020) | Michaelson (2017) |
| Griffin (2006) | Ritchey *et al.* (1957) |
| Gutmann and Rakusin (1994) | Yap (1995) |
| Ito *et al.* (1998) |  |
| **In vitro (n=6)** | |
| Anton *et al.* (2021) | Magne *et al.* (2012) |
| Kim *et al.* (2021) | Naka *et al.* (2018) |
| Lin *et al.* (2013) | Shi *et al.* (2021) |

# References

Abbott PV (2001) Managing cracked teeth with pulpitis. *Journal of Dental Research* **80,** 980-80.

Abbott PV, Leow N (2009) Predictable management of cracked teeth with reversible pulpitis. *Australian Dental Journal* **54,** 306-15.

Abou-Rass M (1983) Crack lines: the precursors of tooth fractures - their diagnosis and treatment. *Quintessence International, Dental Digest* **14,** 437-47.

Anton YOC, Bijelic-Donova J, Saratti CM *et al.* (2021) The influence of FRC base and bonded CAD/CAM resin composite endocrowns on fatigue behavior of cracked endodontically-treated molars. *Journal of the Mechanical Behavior of Biomedical Materials* **121,** 104647.

Banerji S, Mehta SB, Kamran T, Kalakonda M, Millar BJ (2014) A multi-centred clinical audit to describe the efficacy of direct supra-coronal splinting - A minimally invasive approach to the management of cracked tooth syndrome. *Journal of Dentistry* **42,** 862-71.

Batalha-Silva S, Gondo R, Stolf SC, Baratieri LN (2014) Cracked tooth syndrome in an unrestored maxillary premolar: a case report. *Operative Dentistry* **39,** 460-8.

Bearn DR, Saunders EM, Saunders WP (1994) The bonded amalgam restoration - A review of the literature and report of its use in the treatment of four cases of cracked-tooth syndrome. *Quintessence International* **25,** 321-26.

Brynjulfsen A, Fristad I, Grevstad T, Hals-Kvinnsland I (2002) Incompletely fractured teeth associated with diffuse longstanding orofacial pain: diagnosis and treatment outcome. *International Endodontic Journal* **35,** 461-6.

Cameron CE (1976) The cracked tooth syndrome: additional findings. *Journal of the American Dental Association* **93,** 971-75.

Chana H, Kelleher M, Briggs P, Hooper R (2000) Clinical evaluation of resin-bonded gold alloy veneers. *The Journal of Prosthetic Dentistry* **83,** 294-300.

Chen Y-T, Hsu T-Y, Liu H, Chogle S (2021) Factors Related to the Outcomes of Cracked Teeth after Endodontic Treatment. *Journal of Endodontics* **47,** 215-20.

Davis MC, Shariff SS (2019) Success and Survival of Endodontically Treated Cracked Teeth with Radicular Extensions: A 2- to 4-year Prospective Cohort. *Journal of Endodontics* **45,** 848-55.

Davis R, Overton JD (2000) Efficacy of bonded and nonbonded amalgam in the treatment of teeth with incomplete fractures. *Journal of the American Dental Association* **131,** 469-78.

de Toubes KMS, Maia LM, Goulart LC *et al.* (2020) Optimization of results for cracked teeth using CAD-CAM system: A case series. *Iranian Endodontic Journal* **15,** 57-63.

de Toubes KMS, Soares CJ, Soares RV *et al.* (2022) The Correlation of Crack Lines and Definitive Restorations with the Survival and Success Rates of Cracked Teeth: A Long-term Retrospective Clinical Study. *Journal of Endodontics* **48,** 190-99.

Dow DM (2016) Outcome of endodontically treated cracked teeth (Master's Thesis) Bethesda, Maryland, USA: Uniformed Services University of the Health Sciences.

Dutner JM, Herold RW, Wilson JP *et al.* (2020) Fracture necrosis A risk indicator for tooth loss. *Journal of the American Dental Association* **151,** 454-63.

Ehrmann EH, Tyas MJ (1990) Cracked tooth syndrome: diagnosis, treatment and correlation between symptoms and post-extraction findings. *Australian Dental Journal* **35,** 105-12.

Fawzy M, Alhadainy HA, Salah-Uddin M, Abdulrab S (2020) Management of cracked tooth using simvastatin as intracanal medicament. *Clinical case reports* **8,** 3050-53.

Ferracane JL, Hilton TJ, Funkhouser E *et al.* (2022) Outcomes of treatment and monitoring of posterior teeth with cracks: three-year results from the National Dental Practice-Based Research Network. *Clinical Oral Investigations* **26,** 2453-63.

Griffin JD, Jr. (2006) Efficient, conservative treatment of symptomatic cracked teeth. *Compendium of Continuing Education in Dentistry* **27,** 93-112.

Guthrie RC, DiFiore PM (1991) Treating the cracked tooth with a full crown. *Journal of the American Dental Association* **122,** 71-3.

Gutmann JL, Rakusin H (1994) Endodontic and restorative management of incompletely fractured molar teeth. *International Endodontic Journal* **27,** 343-8.

Hilton TJ, Funkhouser E, Ferracane JL *et al.* (2020a) Symptom changes and crack progression in untreated cracked teeth: One-year findings from the National Dental Practice-Based Research Network. *Journal of Dentistry* **93,** 103269.

Hilton TJ, Funkhouser E, Ferracane JL *et al.* (2020b) Recommended treatment of cracked teeth: Results from the National Dental Practice-Based Research Network. *The Journal of Prosthetic Dentistry* **123,** 71-78.

Homewood CIK (1998) Cracked tooth syndrome - Incidence, clinical findings and treatment. *Australian Dental Journal* **43,** 217-22.

Ito K, Nanba K, Akashi T, Murai S (1998) Incomplete fractures in intact bilateral maxillary first molars: a case report. *Quintessence International* **29,** 243-8.

Jun M-K, Park S-W, Lee E-S, Kim B-R, Kim B-I (2019) Diagnosis and management of cracked tooth by quantitative light-induced fluorescence technology. *Photodiagnosis and Photodynamic Therapy* **26,** 324-26.

Kanamaru J, Tsujimoto M, Yamada S, Hayashi Y (2017) The clinical findings and managements in 44 cases of cracked vital molars. *Journal of Dental Sciences* **12,** 291-95.

Kang SH, Kim BS, Kim Y (2016) Cracked Teeth: Distribution, Characteristics, and Survival after Root Canal Treatment. *Journal of Endodontics* **42,** 557-62.

Kim S-Y, Kim S-H, Cho S-B, Lee G-O, Yang S-E (2013) Different treatment protocols for different pulpal and periapical diagnoses of 72 cracked teeth. *Journal of Endodontics* **39,** 449-52.

Kim SY, Kim BS, Kim H, Cho SY (2021) Occlusal stress distribution and remaining crack propagation of a cracked tooth treated with different materials and designs: 3D finite element analysis. *Dental Materials* **37,** 731-40.

Krell KV, Caplan DJ (2018) 12-month Success of Cracked Teeth Treated with Orthograde Root Canal Treatment. *Journal of Endodontics* **44,** 543-48.

Krell KV, Rivera EM (2007) A six year evaluation of cracked teeth diagnosed with reversible pulpitis: treatment and prognosis. *Journal of Endodontics* **33,** 1405-7.

Lee J, Kim S, Kim E, Kim KH, Kim ST, Jeong Choi Y (2021a) Survival and prognostic factors of managing cracked teeth with reversible pulpitis: A 1- to 4-year prospective cohort study. *International Endodontic Journal* **54,** 1727-37.

Lee TY, Yang SE, Kim HM, Kye MJ (2021b) Characteristics, Treatment, and Prognosis of Cracked Teeth: A Comparison with Data from 10 Years Ago. *European Journal of Dentistry* **15,** 694-701.

Leong DJX, de Souza NN, Sultana R, Yap AU (2020) Outcomes of endodontically treated cracked teeth: a systematic review and meta-analysis. *Clinical Oral Investigations* **24,** 465-73.

Liao WC, Tsai YL, Chen KL *et al.* (2022) Cracked teeth: Distribution and survival at 6 months, 1 year and 2 years after treatment. *Journal of the Formosan Medical Association* **121,** 247-57.

Liebenberg WH (1996) Use of resin-bonded partial coverage ceramic restorations to treat incomplete fractures in posterior teeth: a clinical report. *Quintessence International* **27,** 739-47.

Lin CL, Chang YH, Hsieh SK, Chang WJ (2013) Estimation of the failure risk of a maxillary premolar with different crack depths with endodontic treatment by computer-aided design/computer-aided manufacturing ceramic restorations. *Journal of Endodontics* **39,** 375-79.

Liu HH, Sidhu SK (1995) Cracked teeth-treatment rationale and case management: Case reports. *Quintessence International* **26,** 485-92.

Liu SQ, Chen X, Wang XX, Liu W, Zhou X, Wang X (2021) Outcomes and prognostic factors of apical periodontitis by root canal treatment and endodontic microsurgery-a retrospective cohort study. *Annals of Palliative Medicine* **10,** 5027-45.

Lu Y, Wu N, Ma B, Qin F (2021) Effect of Root Canal Therapy Combined with Full Crown Restoration on the Level of Inflammatory Factors and Chewing Function in Patients with Cracked Teeth and Chronic Pulpitis. *Evidence-Based Complementary and Alternative Medicine* **2021,** 3299349.

Magne P, Boff LL, Oderich E, Cardoso AC (2012) Computer-aided-design/computer-assisted-manufactured adhesive restoration of molars with a compromised cusp: effect of fiber-reinforced immediate dentin sealing and cusp overlap on fatigue strength. *Journal of Esthetic and Restorative Dentistry* **24,** 135-46.

Mahgoli HA, Arshad M, Rasouli K (2019) Restoration of endodontically treated cracked maxillary teeth: A case series. *Clinical Case Reports* **7,** 1951-56.

Malentacca A, Zaccheo F, Scialanca M, Fordellone F, Rupe C, Lajolo C (2021) Repair of teeth with cracks in crowns and roots: An observational clinical study. *International Endodontic Journal* **54,** 1738-53.

Marchan SM, Eder A, Marchan QM, Coldero L, Choon AT, Smith WA (2013) A preliminary evaluation into the performance of posterior resin bonded cast metal restorations (adhesive onlays). *The European Journal of Prosthodontics and Restorative Dentistry* **21,** 24-8.

Michaelson PL (2015) A Novel Treatment for Propagated Crown Fractures. *Journal of Endodontics* **41,** 130-34.

Michaelson PL (2017) Long-term Evaluation of Fracture Removal Treatment for Propagated Crown Fractures. *Journal of Endodontics* **43,** 1214-17.

Naka O, Millar BJ, Sagris D, David C (2018) Do composite resin restorations protect cracked teeth? An in-vitro study. *British Dental Journal* **225,** 223-28.

Ng YL, Mann V, Gulabivala K (2011a) A prospective study of the factors affecting outcomes of non-surgical root canal treatment: part 2: tooth survival. *International Endodontic Journal* **44,** 610-25.

Ng YL, Mann V, Gulabivala K (2011b) A prospective study of the factors affecting outcomes of nonsurgical root canal treatment: part 1: periapical health. *International Endodontic Journal* **44,** 583-609.

Nguyen Thi W, Jansson L (2021) Survival rate after endodontic treatment in general dentistry for cracked teeth with different coronal restorations. *Acta Odontologica Scandinavica* **79,** 256-61.

Olivieri JG, Elmsmari F, Miro Q *et al.* (2020) Outcome and Survival of Endodontically Treated Cracked Posterior Permanent Teeth: A Systematic Review and Meta-analysis. *Journal of Endodontics* **46,** 455-63.

Opdam NJM, Roeters FJM (2003) The effectiveness of bonded composite restorations in the treatment of painful, cracked teeth: Six-month clinical evaluation. *Operative Dentistry* **28,** 327-33.

Opdam NJM, Roeters JJM, Loomans BAC, Bronkhorst EM (2008) Seven-year clinical evaluation of painful cracked teeth restored with a direct composite restoration. *Journal of Endodontics* **34,** 808-11.

Ritchey B, Mendenhall R, Orban B (1957) Pulpitis resulting from incomplete tooth fracture. *Oral Surgery, Oral Medicine, Oral Pathology* **10,** 665-70.

Roh B-D, Lee Y-E (2006) Analysis of 154 cases of teeth with cracks. *Dental Traumatology* **22,** 118-23.

Shi R, Meng X, Feng R *et al.* (2021) Stress Distribution and Fracture Resistance of repairing Cracked Tooth with Fiber-reinforced Composites and Onlay. *Australian Endodontic Journal***,** (In Press).

Signore A, Benedicenti S, Covani U, Ravera G (2007) A 4- to 6-year retrospective clinical study of cracked teeth restored with bonded indirect resin composite onlays. *The International Journal of Prosthodontics* **20,** 609-16.

Sim IGB, Lim T-S, Krishnaswamy G, Chen N-N (2016) Decision Making for Retention of Endodontically Treated Posterior Cracked Teeth: A 5-year Follow-up Study. *Journal of Endodontics* **42,** 225-9.

Tan L, Chen NN, Poon CY, Wong HB (2006) Survival of root filled cracked teeth in a tertiary institution. *International Endodontic Journal* **39,** 886-9.

Wu SW, Lew HP, Chen NN (2019) Incidence of Pulpal Complications after Diagnosis of Vital Cracked Teeth. *Journal of Endodontics* **45,** 521-25.

Yap AU (1995) Cuspal coverage with resin-bonded metal onlays. *Dental Update* **22,** 403-6.
