# Supplementary material for: Treatment of cracked teeth: A comprehensive narrative review
Source: Clin Exp Dent Res. 2022 Jul 9;8(5):1218–48. doi: 10.1002/cre2.617 (PMC9562569; doi:10.1002/cre2.617)
Supplement: Supplementary file 2 — Supporting information. [file CRE2-8-1218-s001.docx]

| **SUPPORTING INFORMATION 2.**  Studies excluded at full text stage, with reasons for exclusion | |
| --- | --- |
| **Narrative reviews (n=16)** | |
| Attin (2017) | Pai and Nivedhitha (2020) |
| Bailey (2020) | Rosen (1982) |
| Bhanderi (2021) | Saatwika *et al.* (2020) |
| Braly and Maxwell (1981) | Silvestri and Singh (1978) |
| Geurtsen and García-Godoy (1999) | Swepston and Miller (1986) |
| Geurtsen *et al.* (2003) | West (2002) |
| Longridge and Youngson (2019) | Zimet (1998) |
| Mamoun and Napoletano (2015) | Zimet and Endo (2000) |
| **Not relevant to treatment (n=9)** | |
| Berman and Kuttler (2010) | Ricucci *et al.* (2015) |
| Cameron (1964) | Snyder (1976) |
| Chaponda (1989) | Turp and Gobetti (1996) |
| DiAngelis (1997) | Yang *et al.* (2017) |
| Hiatt (1973) |  |
| **Commentaries (n=5)** | |
| Brignardello-Petersen (2018) | George (2021) |
| Brignardello-Petersen (2020) | Holme (2021) |
| Fain (2021) |  |
| **Opinion articles (Technique description) (n=4)** | |
| Agar and Weller (1988) | Li *et al.* (2015) |
| Clark and Caughman (1984) | Lin *et al.* (2009) |
| **Case reports with no follow-up (n=3)** | |
| Geurtsen (1992) | Trushkowsky (1991) |
| Liebenberg (1995) |  |
| **Not relevant to cracked teeth (n=2)** | |
| Chong (1989) | Göransson *et al.* (2021) |
| **Non-english language (n=1)** | |
| Nie *et al.* (2015) | |

# References

Agar JR, Weller RN (1988) Occlusal adjustment for initial treatment and prevention of the cracked tooth syndrome. *The Journal of Prosthetic Dentistry* **60,** 145-7.

Attin T (2017) The problem of longitudinal tooth fractures. *Acta Stomatologica Croatica* **51,** 249.

Bailey O (2020) Cracked tooth syndrome management part 2: Integrating the old with the new. *Dental Update* **47,** 570-82.

Berman LH, Kuttler S (2010) Fracture Necrosis: Diagnosis, Prognosis Assessment, and Treatment Recommendations. *Journal of Endodontics* **36,** 442-46.

Bhanderi S (2021) Facts About Cracks in Teeth. *Primary Dental Journal* **10,** 20-27.

Braly BV, Maxwell EH (1981) Potential for tooth fracture in restorative dentistry. *The Journal of Prosthetic Dentistry* **45,** 411-14.

Brignardello-Petersen R (2018) Almost one-half of cracked posterior teeth were symptomatic, and pain to cold was the most common finding. *Journal of the American Dental Association* **149,** E118-E18.

Brignardello-Petersen R (2020) There seems to be a high average survival rate of cracked teeth that have undergone endodontic treatment. *Journal of the American Dental Association* **151,** E52-E52.

Cameron CE (1964) Cracked-tooth syndrome. *Journal of the American Dental Association* **68,** 405-11.

Chaponda A (1989) Incomplete tooth fracture - A case-report. *Journal of Dental Research* **68,** 651-51.

Chong BS (1989) Bilateral cracked teeth: a case report. *International Endodontic Journal* **22,** 193-96.

Clark LL, Caughman WF (1984) Restorative treatment for the cracked tooth. *Operative Dentistry* **9,** 136-42.

DiAngelis AJ (1997) The lingual barbell: a new etiology for the cracked-tooth syndrome. *Journal of the American Dental Association* **128,** 1438-9.

Fain WD (2021) Tooth Fractures and Cracks. *Journal of the American Dental Association* **152,** 423-24.

George R (2021) What is the evidence of outcomes of endodontically treated cracked teeth? *Evidence-Based Dentistry* **22,** 22-23.

Geurtsen W (1992) The cracked-tooth syndrome: Clinical features and case reports. *International Journal of Periodontics and Restorative Dentistry* **12,** 395-405.

Geurtsen W, García-Godoy F (1999) Bonded restorations for the prevention and treatment of the cracked-tooth syndrome. *American Journal of Dentistry* **12,** 226-70.

Geurtsen W, Schwarze T, Gunay H (2003) Diagnosis, therapy, and prevention of the cracked tooth syndrome. *Quintessence International* **34,** 409-17.

Göransson H, Lougui T, Castman L, Jansson L (2021) Survival of root filled teeth in general dentistry in a Swedish county: a 6-year follow-up study. *Acta Odontologica Scandinavica* **79,** 396-401.

Hiatt WH (1973) Incomplete crown-root fracture in pulpal-periodontal disease. *Journal of Periodontology* **44,** 369-79.

Holme W (2021) Does bidirectional splinting improve outcomes in cracked teeth with reversible pulpitis? *Evidence-Based Dentistry* **22,** 152-53.

Li BL, Ying C, Li QL (2015) Mussel adhesive protein coating: A potential therapeutic method for self-healing of cracked teeth. *Dental Hypotheses* **6,** 82-85.

Liebenberg WH (1995) Esthetics in the Cracked Tooth Syndrome: Steps to Success Using Resin‐Bonded Ceramic Restorations. *Journal of Esthetic and Restorative Dentistry* **7,** 155-66.

Lin YX, Zheng R, He H, Du HL, Lin YH (2009) Application of biomimetic mineralization: A prophylactic therapy for cracked teeth? *Medical Hypotheses* **73,** 493-94.

Longridge NN, Youngson CC (2019) Dental Pain: Dentine Sensitivity, Hypersensitivity and Cracked Tooth Syndrome. *Primary Dental Journal* **8,** 44-51.

Mamoun JS, Napoletano D (2015) Cracked tooth diagnosis and treatment: An alternative paradigm. *European Journal of Dentistry* **9,** 293-303.

Nie EM, Jiang R, Zhang CY, Zeng JD, Tan JZ (2015) Temporary protection of cracked teeth using orthodontic band and resin temporary crown. *Chinese Journal of Tissue Engineering Research* **19,** 4805-09.

Pai S, Nivedhitha MS (2020) Diagnosis and Management of Cracked Tooth-Decision Analysis. *Bioscience Biotechnology Research Communications* **13,** 457-63.

Ricucci D, Siqueira JF, Jr., Loghin S, Berman LH (2015) The cracked tooth: histopathologic and histobacteriologic aspects. *Journal of Endodontics* **41,** 343-52.

Rosen H (1982) Cracked tooth syndrome. *The Journal of Prosthetic Dentistry* **47,** 36-43.

Saatwika L, Prakash V, Malarvizhi D, Subbiya A (2020) A review on cracked tooth syndrome. *Indian Journal of Forensic Medicine and Toxicology* **14,** 1119-22.

Silvestri AR, Jr., Singh I (1978) Treatment rationale of fractured posterior teeth. *Journal of the American Dental Association* **97,** 806-10.

Snyder DE (1976) The cracked-tooth syndrome and fractured posterior cusp. *Oral Surgery, Oral Medicine, and Oral Pathology* **41,** 698-704.

Swepston JH, Miller AW (1986) The incompletely fractured tooth. *The Journal of Prosthetic Dentistry* **55,** 413-16.

Trushkowsky R (1991) Restoration of a cracked tooth with a bonded amalgam. *Quintessence International* **22,** 397-400.

Turp JC, Gobetti JP (1996) The cracked tooth syndrome: An elusive diagnosis. *Journal of the American Dental Association* **127,** 1502-07.

West JD (2002) The cracked tooth syndrome. *Dentistry Today* **21,** 88-97.

Yang S-E, Jo AR, Lee H-J, Kim S-Y (2017) Analysis of the characteristics of cracked teeth and evaluation of pulp status according to periodontal probing depth. *BMC Oral Health* **17,** 135.

Zimet PO (1998) Cracked tooth syndrome. *Australian Endodontic Journal* **24,** 33-37.

Zimet PO, Endo C (2000) Preservation of the roots--management and prevention protocols for cracked tooth syndrome. *Annals of the Royal Australasian College of Dental Surgeons* **15,** 319-24.
